# Social Support and Network Formation in a Small-Scale Horticulturalist Population

**DOI:** 10.1038/s41597-022-01516-x

**Published:** 2022-09-15

**Authors:** Cohen R. Simpson

**Affiliations:** 1 Department of Methodology, The London School of Economics and Political Science, London, UK; 2 Nuffield College, University of Oxford, Oxford, UK

**Keywords:** Behavioural ecology, Biological anthropology, Complex networks, Sociology

## Abstract

Evolutionary studies of cooperation in traditional human societies suggest that helping family and responding in kind when helped are the primary mechanisms for informally distributing resources vital to day-to-day survival (e.g., food, knowledge, money, childcare). However, these studies generally rely on forms of regression analysis that disregard complex interdependences between aid, resulting in the implicit assumption that kinship and reciprocity drive the emergence of entire networks of supportive social bonds. Here I evaluate this assumption using individual-oriented simulations of network formation (i.e., Stochastic Actor-Oriented Models). Specifically, I test standard predictions of cooperation derived from the evolutionary theories of kin selection and reciprocal altruism alongside well-established sociological predictions around the self-organisation of asymmetric relationships. Simulations are calibrated to exceptional public data on genetic relatedness and the provision of tangible aid amongst all 108 adult residents of a village of indigenous horticulturalists in Nicaragua (11,556 ordered dyads). Results indicate that relatedness and reciprocity are markedly less important to whom one helps compared to the supra-dyadic arrangement of the tangible aid network itself.

## Introduction

Human evolutionary research has historically conceptualised social support as a purely dyadic phenomenon (e.g., see Refs. ^1–16^). That is, given some trait pertaining to two persons *i* and *j* — e.g., their genetic relatedness, history of helping each other, physical proximity, or difference in wealth — does *i* help *j*? Both elegant and tractable, this dyad-centric view of social support evokes classic theoretical models of cooperation as a “Prisoner’s Dilemma” within a void consisting only of ego (*i*) and alter (*j*)^17^. Yet it also belies the fact that aid relationships (i.e., who helps who) constitute complex networks of supportive social bonds that emanate throughout entire human communities.

Members of such networks may, in principle, unilaterally help whomever they wish. And their decisions to help — or to not help — specific others comprise a dynamic, supra-dyadic relational context that shapes one’s plausible set of aid targets at the micro level^18–22^. Put simply, in social support networks, aid is targeted and interdependent across dyads such that the patterning of cooperation among multiple alters jointly affects whom any one network member helps. This sociocentric (i.e., whole network) view of social support is distinct from the perspective taken by evolutionary graph theorists who study the emergence of cooperation **on** network structure and other spatial substrates (e.g., square grids) that may be fixed or dynamic (e.g., see Refs. ^23–25^). And it is distinct from the perspective taken by analysts of egocentric (i.e., personal) networks who study how the arrangement of intimate relationships exclusively between one’s closest contacts (e.g., the extent to which one’s friends are also friends) eases access to help (e.g., see Martí, Bolíbar, and Lozares^26^).

Differences between the dyad-centric and the sociocentric perspectives on social support are not merely cosmetic. Indeed, the dyad-centric stance of human evolutionary research has led to a situation wherein the relational context of helping behaviour is underexplored. And this has, in turn, impaired understanding of the relative importance of fundamental evolutionary mechanisms to the structuring of cooperative relationships in human communities.

Specifically, human evolutionary research on helping behaviour generally takes the theories of kin selection and reciprocal altruism as lodestars. In so doing, sociometric data from subsistence societies across the globe have been used to investigate whether consanguinity (i.e., genetic kinship) and reciprocity govern aid unconditionally and in relation to multiple social and demographic factors. These include affinity (i.e., marriage-based kinship), physical proximity, relative need, homophily (e.g., based on age and gender), social closeness, friendship, religiosity, reputation, conflict, status, and anthropometric measurements such as size, height, and strength. And, on balance, evidence^1–10,13,14,16,27–33^ suggests that helping family and responding in kind when helped are the primary mechanisms by which humans informally distribute resources vital to day-to-day survival (e.g., advice, information, food, money, durables, and physical assistance).

However, despite laudable exceptions^2,7,15,28–34^ and perhaps due to the influence of methodological trends in the wider behavioural ecology literature on relationships between animals (see Refs. ^35–37^), human evolutionary studies of real helping behaviour have typically relied on non-network methods — namely, monadic regression, dyadic regression, and permutation tests (e.g., see Refs. ^1–3,5,6,8–14,16,27^). Respectively, these techniques treat the supra-dyadic structure of social support networks as ignorable, reducible to dyads, or a nuisance to be corrected for^38^. Yet, sociocentric research by sociologists^39–49^ firmly establishes that humans create and maintain relationships in accordance with factors intrinsic to the supra-dyadic arrangement of network structure itself (e.g., processes of degree-reinforcement and group formation involving at least three persons). And this sociological research makes clear that network-structure-related dynamics can operate simultaneously and independently of non-network factors (e.g., age and kinship).

Ultimately, reliance on methods that disregard complex interdependences between aid obscures the extent to which helping family and responding in kind when helped outrank the dynamics of the cooperative system within which decisions to assist specific individuals take place. This uncertainty represents a substantial gap in our scientific understanding of altruism. Accordingly, here I tackle a major point of interest in evolutionary anthropology and human behavioural ecology^50^ specifically through the lens of the sociology of social networks^18,21,51^, asking:

**RQ**: How important is helping family and responding in kind when helped relative to supra-dyadic, network-structure-related constraints on the provision of aid?

### The Current Study

To answer my research question, I use Koster’s^27^ recently-released cross-sectional data on genetic relatedness and the habitual provision of tangible aid (e.g., firewood, food, valuable items, and/or physical assistance). Re-analysed here due to their exceptional detail and measurement quality in addition to their broad relevance to the scientific community (see *Methods*), these data were collected in 2013 and concern a complete population. Specifically, they cover all 108 adult (18+) residents (11,556 ordered dyads) of the 32 households of Arang Dak — a remote village of 279 indigenous Mayangna and Miskito swidden (i.e., “slash-and-burn”) horticulturalists. Arang Dak sits on the Lakus River in Nicaragua’s Bosawás Biosphere Reserve, a neotropical forest in the Department of Jinotega.

In total, the tangible aid network that I analyse — i.e., *x*(*t*_2013_)— consists of 1,485 asymmetric aid relationships between the adult residents of Arang Dak. Of the 1,485 aid relationships, 1,422 are verified by the source **and** the recipient of help. That is, *x*_*ij*_(*t*_2013_) = 1 if villager *i* reported in 2013 that they give tangible aid to villager *j* at least once per month and villager *j* reported in 2013 that they receive tangible aid from villager *i* at least once per month. Still, note that Koster’s^27^ data document self-reported resource flows as opposed to observed transfers. Named sources and targets of aid are based on the village roster — not freely recalled from memory. See *Methods* for a summary of the data and details on the measurement of the network and kinship.

#### Modelling Strategy

**Fig. 1:**
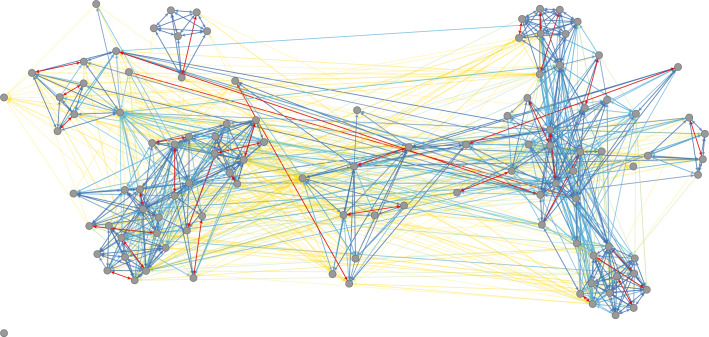
Kinship and tangle aid in Arang Dak. Each arc (i.e., directed relationship) indicates the provision of tangible aid by some villager *i* to some other villager *j* (108 villagers; 1,485 arcs). Arcs are coloured to reflect whether the source and target of aid are “close kin” (i.e., consanguineal relatedness or affinal relatedness ≥ 0.125). Red arcs emanate from spouses (i.e., affinal relatedness = 1.0). Dark-blue arcs emanate from primary kin — i.e., relatives with a consanguineal or affinal relatedness equal to 0.5 (e.g., full-siblings, parent and child, wife and brother-in-law, husband and mother-in-law). Lighter-blue arcs and green arcs emanate from near kin (i.e., 0.375 ≥ consanguineal/affinal relatedness ≥ 0.125). And yellow arcs emanate from kin who are not close — either genetically or through marriage (i.e., consanguineal/affinal relatedness < 0.125) — or emanate from residents of Arang Dak who are not related (i.e., consanguineal/affinal relatedness = 0). See also “Rationale for Network Diagram Construction” in *Methods*.

To analyse tangible aid in relation to supra-dyadic network structure (Fig. 1), I use generative network models following Redhead and von Rueden^32^ and von Rueden *et al*.^33^, amongst other human evolutionary scientists^2,7,15,28–34^. Specially, I rely on Stochastic Actor-Oriented Models (SAOMs) which are used for observational (i.e., non-causal) analyses of the temporal evolution of networks.

Put simply, SAOMs are akin to multinomial logistic regression. More formally, SAOMs are simulations of individual network members’ choices between **outgoing** relationships with different rewards and costs. These simulations are calibrated or “tuned” to the observed network data. That is, conditional on *x* (i.e., the observed states of a dynamic network), SAOMs simulate network evolution between successive observations or “snapshots” of the network at $M$ discrete time points — i.e., $x\left({t}_{m}\right)\to x\left({t}_{m+1}\right)$ — as a continuous-time, Markovian process of repeated, asynchronous, and sequential tie changes. The Markovian process is defined on the space of all possible directed graphs for a set of *N* = {1, …, *n*} network members^40,42,44,52–55^.

SAOMs decompose change between successive network observations into its smallest possible unit. Specifically, “change” means creating one outgoing tie if it does not exist, dropping one outgoing tie if it does, or doing nothing (i.e., maintaining the status quo network). More formally, during a SAOM simulation, focal actors *i* (ego) myopically modify just one of their outgoing relationships with some alter *j* in the set of network members *N* (i.e., *j* ∈ *N*, *j* ≠ *i*). The change made by *i* is the change that maximises a utility or “evaluation” function. In this respect, the evaluation function captures the “attractiveness”^44^ of tie changes — where “attraction” means “…something like ‘sending a tie to [an actor *j*] with a higher probability if all other circumstances are equal.’” (Snijders and Lomi^56^, p. 5).

The evaluation function itself is a weighted sum of parameter estimates $\widehat{\beta }$ and their associated covariates *k* (i.e., SAOM “effects”^44^) plus a Gumbel-distributed variable used to capture random influences^55^. The simulated tie changes or “ministeps”^44^ made by *i* shift the network between adjacent (unobserved) states. These states differ, at most, by the presence/absence of a single tie^40,42^. And the probabilities of the ministeps — a large number of which are required to bring one observation of the network to the next (i.e., $x\left({t}_{m}\right)\to x\left({t}_{m+1}\right)$) — are given by a multinomial logit which uses the evaluation function as the linear predictor.

Each covariate *k* used to specify the evaluation function summarises some structural (i.e., purely network-related) feature or non-structural feature of *i*’s immediate (i.e., local) network — e.g., the sum of the in-degrees of *i*’s alters, the number of reciprocated dyads that *i* is embedded in, or *i*’s number of outgoing ties weighted by genetic relatedness. These features correspond to theoretical mechanisms of interest (e.g., preferential attachment, reciprocal altruism, or kin selection) and generally take the form of unstandardised sums.

SAOM parameter estimates $\widehat{\beta }$ (log odds ratios) summarise the association between the covariates and the **simulated** tie changes or “ministeps”. Specifically, should a focal actor *i* have the opportunity to make a ministep in departure from some current (i.e., status-quo) network state *x* in transit to a new network state *x*^±*ij*^ — i.e., the adjacent network defined by *i*’s addition/subtraction of the tie *x*_*ij*_ to/from *x* — ${\widehat{\beta }}_{k}$ is the log odds of choosing between two different versions of *x*^±*ij*^ in relation to some covariate *k*. For example, ${\widehat{\beta }}_{{\rm{Reciprocity}}}=1.7$ would indicate that the log odds of *i* creating and maintaining the supportive relation *x*_*ij*_ is, conditional on the other covariates *k*, larger by 1.7 when *x*_*ij*_ reciprocates a tie (i.e., *x*_*ji*_) compared to when *x*_*ij*_ does not reciprocate a tie (i.e., reciprocated ties are more “attractive”). In contrast, ${\widehat{\beta }}_{{\rm{Reciprocity}}}=-1.7$ would indicate that the log odds of *x*_*ij*_ is, conditional on the other effects, smaller by −1.7 when *x*_*ij*_ reciprocates a tie compared to when *x*_*ij*_ does not reciprocate a tie (i.e., reciprocated ties are less “attractive”).

Given the longitudinal nature of the model, the gain in the evaluation function for a ministep is determined by the *difference* Δ ﻿in the value of the statistic *s* for a covariate *k* — i.e., Δ_*k*,*ij*_(*x*, *x*^±*ij*^) = *s*_*k,i*_(*x*^±*ij*^) − *s*_*k,i*_(*x*) — incurred through the addition/subtraction of *x*_*ij*_ to/from *x* (see Block *et al*.^42^ and Ripley *et al*.^44^ on “change statistics”). Accordingly, ${\widehat{\beta }}_{{\rm{Reciprocity}}}=1.7$, for example, is the value that *x*_*ij*_ positively contributes to the evaluation function when *x*_*ij*_ increases the network statistic *s*_*k,i*_(*x*) underlying the *Reciprocity* effect by the value of one (i.e., Δ_Reciprocity,*ij*_ (*x*, *x*^±*ij*^) = *s*_Reciprocity,*i*_(*x*^±*ij*^) − *s*_Reciprocity,*i*_ (*x*) = 1 − 0 = 1).

The probabilities of network members being selected for a ministep is governed by a separate “rate” function. And the baseline rate parameter *λ* is a kind of intercept for the amount of network change between successive observations of the analysed network. Larger baseline rates indicate that, on average, more simulated tie changes were made to bring one observation of the network to the next (i.e., $x\left({t}_{m}\right)\to x\left({t}_{m+1}\right)$).

However, as the data from Nicaragua are from a single point in time (i.e., 2013), I use the *cross-sectional* or *stationary* Stochastic Actor-Oriented Model (cf. von Rueden *et al*.^33^). Accordingly, Arang Dak’s tangible aid network is assumed to be in “short-term dynamic equilibrium.” As Snijders and Steglich^40^ (p. 265) discuss in detail, “this ‘short-term equilibrium’ specification of the SAOM is achieved by requiring that the observed network is both the centre and the starting value of a longitudinal network evolution process in which the number of change opportunities per actor [i.e., *λ*] is fixed to some high (but not too high) value.”

Practically speaking, this means that the cross-sectionally observed network is used as the beginning **and** the target state for a SAOM simulation — i.e., $x\left({t}_{2013}\right)\to x\left({t}_{2013}\right)$ — during which actors are allowed to make, on average, very many changes (i.e., *λ*) to their portfolio of outgoing ties. These simulated tie changes produce a distribution of synthetic networks with properties that are, on average, similar to those of the cross-sectionally observed network in a converged SAOM — where the target properties correspond to the researcher-chosen SAOM effects *k*. Put simply, “[cross-sectional] SAOMs assume that the network structure, although changing, is in a stochastically stable state.” (Krause, Huisman, and Snijders^57^, p. 36–37). Thus, the estimated parameters $\widehat{\beta }$ continue to summarise the rules by which ministeps unfold. However, the asynchronous, sequential, simulated tie changes, in a sense, “cancel out” and thus hold the network in “short-term dynamic equilibrium”^40,42^. Formally, the cross-sectional SAOM is defined as a stationary distribution of a Markov Chain with transition probabilities given by the multinomial logit used to model change between adjacent network states^40,42^.

The rate parameter *λ* is fixed at 36 for my analysis. The value of 36 is the maximum observed out-degree in the source-recipient-verified tangible aid network *x*(*t*_2013_). Accordingly, under *λ* = 36, all members of the tangible aid network have, on average, at least one opportunity to modify their entire portfolio of outgoing ties during the simulations. Nevertheless, to ensure the robustness of my results, I also fit a second set of models for which *λ* was fixed to 108 (i.e., thrice the maximum out-degree).

#### Model Specification

To assess the importance of kinship and reciprocity to hypothetical decisions to help others (i.e., ministeps), I use four archetypal specifications of the SAOM’s evaluation function. These model specifications feature nested sets of covariates (i.e., the SAOM “effects”^44^). And, using language found in prior evolutionary studies^3,5^, I refer to these archetypal specifications as the “Conventional Model” (Model 1) of aid, the “Extended Model” (Model 2) of aid, the “Networked Aid Model (Limited)” (Model 3), and the “Networked Aid Model (Comprehensive)” (Model 4).

The first specification (i.e., Model 1) comes from Hackman *et al*.^3^ and Kasper and Borgerhoff Mulder^5^ who respectively label it the “Human Behavioural Ecology” and “Conventional” model. This specification is comprised of just four dyadic covariates — one each for consanguinity (i.e., Wright’s coefficient of genetic relatedness), affinity (i.e., Wright’s coefficient of genetic relatedness between *i*’s *spouse s* and his/her blood relative *j*), the receipt of aid, and geographic distance. The first three covariates are used to test long-standing predictions of helping in order to reap indirect and direct fitness benefits in line with the theories of kin selection and reciprocal altruism (see Refs. ^1,5,27,58,59^ for primers). And the fourth covariate is used to adjust for tolerated scrounging — i.e., what Jaeggi and Gurven^4^ (p. 2) define as aid resulting from one’s inability to monopolise resources due to costs imposed by the resource-poor — where a covariate for distance operationalises pressure to help imposed by those who are spatially close^4^.

The second specification (i.e., Model 2) reflects Kasper and Borgerhoff Mulder’s^5^ and Thomas *et al*.’s^9^ extensions to the conventional model (see also Page *et al*.^16^). Specifically, and following important work by Allen-Arave, Gurven, and Hill^1^, Hooper *et al*.^14^, and Nolin^7^, it is distinguished by nuanced tests of kin selection and reciprocal altruism via interactions between: (*i*) consanguinity and the receipt of aid; (*ii*) consanguinity and relative need; and (*iii*) consanguinity and geographic distance. Furthermore, Kasper and Borgerhoff Mulder’s^5^ and Thomas *et al*.’s^9^ extended model includes covariates for the non-network-related attributes of individuals (e.g., gender, wealth, and physical size), thus adjusting for homophily, trait-based popularity, trait-based activity, and local context (e.g., results from a gift-giving game^9^ or, in the present case, infidelity and discrimination based on skin-tone^27^).

The third specification (i.e., Model 4) is my revision of the second. It is geared to make the relational context of aid explicit. This is done using nine covariates that account for the breadth of sociologists’ contemporary understanding of supra-dyadic interdependence between positive-valence (i.e., not based on disliking or aggression), asymmetric social relationships^39–49^. In keeping with the nature of the SAOM, each of these covariates summarises some structural feature of a villager’s immediate (i.e., local) network (e.g., the number of transitive triads that she is embedded in). Accordingly, each structural covariate is used to capture a form of *self-organisation* — i.e., network formation driven by an individual’s selection of alters in response to network structure itself (Lusher *et al*.^49^, p. 10–11 and 23–27).

Specifically, the covariates added in the third specification reflect predictions derived from three fundamental sociological theories of the emergence of non-romantic relationships. The first is structural balance theory which posits that individuals create and maintain ties that move groups of three people from an intransitive to a transitive state (i.e., transitive closure), the latter of which is understood to be more psychologically harmonious or “balanced” (see Refs. ^39,43,47,48,60–62^ for primers). The second is Simmelian tie theory which posits that, once formed, individuals will maintain relationships embedded in maximally-cohesive groups of three people such that 3-cliques (i.e., fully-reciprocated triads) are resistant to dissolution (see Refs. ^43,48,63^ for primers). The third is social exchange theory (as it relates to structured reciprocity) which posits that individuals will unilaterally give benefits to others in response to benefits received such that indirect reciprocity (i.e., returns to generosity) and generalised reciprocity (i.e. paying-it-forward) in groups of three people encourage cyclic closure — i.e., the simplest form of chain-generalised exchange (see Refs. ^19,20,43^ for primers). Furthermore, the third specification reflects the broad prediction that individuals vary in their propensity to send and receive relationships based on their structural position alone (e.g., popularity-biased attachment) leading to dispersion in the distribution of in-degrees and out-degrees (see Refs. ^39,44,49^ for primers) — especially for ties with an inherent cost to their maintenance^39,42^.

Last, I consider a fourth specification (i.e., Model 3) that uses a subset of the nine network-structure-related covariates included in Model 4. This limited set of structural effects typifies the specifications used in prior human evolutionary studies of empirical help that present generative models of entire networks^2,7,15,28–34^. Specifically, the fourth specification features just three network-structure-related covariates to account for structural balance theory, self-reinforcing in-degree (i.e., popularity-bias), and the interplay between in-degree and out-degree.

Descriptive statistics for the relevant attributes of the 108 residents of Arang Dak appear in Table 1. Formulae used to calculate the network statistics *s*_*k,i*_(*x*) underlying each effect *k* used to specify my SAOMs, alongside verbal descriptions to aid reader interpretation, appear in Online-Only Table 1. See *Methods* for additional rationale behind the third specification.

**Table 1:** Descriptive statistics for the monadic and dyadic attributes of the residents of Arang Dak.

Variable	Description	N	Mean	SD	Median	Minimum	Maximum	Levels
Gender	Female = 1, Male = 0	108	—	—	—	0 (N = 54)	1 (N = 54)	2
Ethnicity	Miskito = 1, Mayangna = 0	108	—	—	—	0 (N = 92)	1 (N = 16)	2
Melanin Index	Measure of skin pigmentation (reflectance spectroscopy) using a resident’s forehead. Higher values indicate darker skin tone.	108	51.55	4.65	50.65	43.40	67	—
Age	Years of age estimated using various methods (e.g., self-reports, government ID, key events).	108	34.48	13.83	31.50	18	75	—
Body Mass Index (BMI)	A resident’s weight (kilograms) divided by their squared height (metres). Weight and height were measured one month before the sociometric interviews.	108	23.91	2.59	23.47	15.89	32.20	—
Household Wealth	Approximate monetary value (Nicaraguan Córdoba) of the key possessions in a resident’s home. Surveyed items include livestock and tools.	108	654.70	995.09	323.03	49	5,259.30	—
Household Size	The number of *adults* living in a resident’s home including the resident him/herself.	108	4.39	2.38	4	2	10	—
Relative Wealth Rank	Asymmetric dyadic variable for Intra-village Household Wealth Rank_*i*_ – Intra-village Household Wealth Rank_*j*_. There are 32 households in Arang Dak.	11,556	—	—	—	−31	31	—
Geographic Distance	Symmetric dyadic variable for distance (metres) between the home of $i$ and the home of $j$.	5,778	518.50	977.16	209.77	0	4,396.11	—
Consanguineal Relatedness	Symmetric dyadic variable for Wright’s coefficient of genetic relatedness (genealogically derived) between $i$ and $j$. Bounded between 0 and 1. Higher values indicate that two individuals are more closely related.	5,778	0.05	0.11	0	0	0.50	—
Affinal Relatedness	Symmetric dyadic variable for Wright’s coefficient of relatedness between $i$’s *spouse* $s$ and $j$.	5,778	0.06	0.13	0	0	1 (Spouses)	—
Godparental Relation	Symmetric dyadic indicator for whether resident *i* is the godparent of resident *j* (or one or more of *j*’s children) or vice versa.	5,778	—	—	—	0 (N = 5,578)	1 (N = 200)	2
Infidelity Relation	Symmetric dyadic indicator for whether resident *i* and resident *j* are members of households with children who are half-siblings as a result of adulterous relations (e.g., *x*_*ij*_ = 1 if resident *i*, or one of *i*’s cohabitants, has an illegitimate child in the household of *j* or vice versa).	5,778	—	—	—	0 (N = 5,720)	1 (N = 58)	2

All Stochastic Actor-Oriented Models were fit using the standardised version (i.e., Z-Score) of Household Wealth (Natural Log *NIO* before standardisation), Household Size, Age, Melanin Index, and BMI by subtracting from each variable its global mean and then dividing by its standard deviation. In total, there were 108 adults, 11,556 ordered dyads, and 5,778 unordered/unique dyads in Arang Dak at the time of data collection in 2013.

#### Model Comparison

Compared to prior human evolutionary research on social support networks, I take two novel approaches to gauging the importance of kinship and reciprocity to help. First, I use a technique^41^ specifically designed to measure the relative importance of individual effects in SAOMs (see *Methods*). And second, I evaluate each specification’s ability to produce synthetic graphs with topologies representative of the structure of the analysed tangible aid network^64^.

Judging model specifications using topological properties reflects one of the core purposes of methods such as the SAOM and the Exponential Random Graph Model (ERGM) — i.e., to explain the emergence of global network structure (see Refs. ^40,42,46,47,49^ also Refs. ^18,48^), not simply the state of individual dyads (i.e., is aid given or not?). Admittedly, explaining global network structure is not a stated primary aim of dyadic-centric or sociocentric studies of help by human evolutionary scientists, including those wherein authors rely on SAOMs or ERGMs^2,7,15,28–34^. Still, topological reproduction is an important, strong test of the relative quality of the four archetypal specifications as each encodes the set of rules presumed to govern network members’ decisions about whom to help.

To clarify, recall that here it is assumed, *a priori*, that network members can, in principle, cooperate with whomever they wish, that their cooperative decisions are intertwined across multiple scales, and that their micro-level decisions ultimately give rise to macro-level patterns of supportive social bonds (see Refs. ^18–22^). The macro-level patterns generated by SAOMs and ERGMs can differ dramatically based on specification^40,46,47,49,64,65^. Thus, the empirical relevance of a candidate model rests with its ability to produce synthetic graphs similar to the observed structure^40,42,46–49,64^. Ultimately, divergence between the real and simulated graphs suggests that a candidate specification is suspect as it does not describe how some network of interest could have formed.

## Results

Parameter estimates $\widehat{\beta }$ (associational; non-causal) from the Conventional and Extended Models, as well as estimates from the limited and comprehensive versions of the Networked Aid Model, appear in Table 2 and Table 3. Note well that results are for cross-sectional SAOMs^40,42^. Thus, estimates relate to a simulation of sequential tie changes wherein network members have, on average, a large, fixed number of opportunities (i.e., *λ*) to modify their portfolio of outgoing relationships. Again, this simulation is “tuned” to the single observation of Arang Dak’s tangible aid network *x*(*t*_2013_), where the cross-sectionally observed network is represented by a probability distribution in the form of a stationary distribution of a dynamic interaction process that is Markovian (Snijders and Steglich^40^, p. 224 and 243; Block *et al*.^42^, p. 208–209). Put alternatively, “…correspondence between the [SAOM] and [the] single-observed network is specified by considering…model parameters for which the observed [network] is in a short-term dynamic equilibrium” (Snijders and Steglich^40^, p. 243). Recall that the rate parameter *λ* is fixed at 36 (Table 2). However, results are robust to a sizeable increase of *λ* to 108 (Table 3).

**Table 2:** Estimated SAOM parameters $\widehat{\beta }$ when the rate parameter *λ* is fixed at 36. Estimates are log odds ratios (e.g., ${\widehat{\beta }}_{{\rm{Reciprocity}}}=1.7$ indicates that the odds of the aid relation *x*_*ij*_ is, conditional on the other effects, larger by a factor of 5.5 (*e*^1.7^) when *x*_*ij*_ reciprocates the tie *x*_*ji*_).

SAOM Effect	Conventional Model (1)			Extended Model (2)			Networked Aid Model (Limited) (3)			Networked Aid Model (Comprehensive) (4)		
	$\widehat{{\boldsymbol{\beta }}}$	${{\boldsymbol{s}}{\boldsymbol{e}}}_{\widehat{{\boldsymbol{\beta }}}}$	*p*	$\widehat{{\boldsymbol{\beta }}}$	${{\boldsymbol{s}}{\boldsymbol{e}}}_{\widehat{{\boldsymbol{\beta }}}}$	*p*	$\widehat{{\boldsymbol{\beta }}}$	${{\boldsymbol{s}}{\boldsymbol{e}}}_{\widehat{{\boldsymbol{\beta }}}}$	*p*	$\widehat{{\boldsymbol{\beta }}}$	${{\boldsymbol{s}}{\boldsymbol{e}}}_{\widehat{{\boldsymbol{\beta }}}}$	*p*
Rate Param. *λ* (Avg. Tie Changes)	36	Fixed	—	36	Fixed	—	36	Fixed	—	36	Fixed	—
Out-degree (Aid Arbitrary People)	−1.558	0.083	0.000	−1.653	0.151	0.000	−1.849	0.169	0.000	−2.570	0.201	0.000
Reciprocity	1.652	0.062	0.000	1.726	0.078	0.000	1.722	0.094	0.000	2.209	0.128	0.000
Relative Wealth Rank	—	—	—	0.011	0.008	0.167	0.009	0.007	0.190	0.008	0.008	0.267
Geographic Distance	−0.062	0.014	0.000	−0.118	0.019	0.000	−0.080	0.020	0.000	−0.063	0.020	0.001
Consanguineal Relatedness	2.847	0.169	0.000	3.312	0.400	0.000	2.420	0.411	0.000	1.846	0.421	0.000
Cons. Rel. × Reciprocity	—	—	—	−1.288	0.407	0.002	−1.180	0.420	0.005	−0.675	0.428	0.115
Cons. Rel. × Relative Wealth Rank	—	—	—	−0.015	0.017	0.373	−0.017	0.017	0.310	−0.013	0.017	0.444
Cons. Rel. × Geographic Distance	—	—	—	0.047	0.061	0.440	−0.021	0.064	0.738	−0.014	0.066	0.837
Affinal Relatedness	2.082	0.145	0.000	2.007	0.156	0.000	1.219	0.163	0.000	0.892	0.169	0.000
Godparental Relation	—	—	—	0.430	0.084	0.000	0.398	0.090	0.000	0.363	0.092	0.000
Infidelity Relation	—	—	—	−0.348	0.213	0.103	−0.246	0.220	0.262	−0.135	0.224	0.547
Household Wealth (Alter)	—	—	—	0.055	0.067	0.410	0.055	0.060	0.360	0.041	0.068	0.550
Household Size (Alter)	—	—	—	−0.005	0.026	0.858	−0.033	0.024	0.183	−0.015	0.027	0.573
Age (Alter)	—	—	—	0.084	0.025	0.001	0.086	0.033	0.008	0.037	0.038	0.331
Gender: Female (Alter)	—	—	—	0.125	0.051	0.015	0.061	0.052	0.246	0.017	0.058	0.764
Ethnicity: Miskito (Alter)	—	—	—	0.034	0.112	0.761	0.057	0.108	0.594	0.084	0.118	0.476
Melanin Index (Alter)	—	—	—	−0.063	0.029	0.029	−0.072	0.027	0.008	−0.036	0.029	0.227
Body Mass Index (Alter)	—	—	—	−0.091	0.026	0.001	−0.037	0.026	0.150	−0.062	0.028	0.027
Household Wealth (Ego)	—	—	—	0.014	0.066	0.838	−0.034	0.059	0.563	−0.043	0.065	0.506
Household Size (Ego)	—	—	—	−0.129	0.027	0.000	−0.118	0.027	0.000	−0.119	0.030	0.000
Age (Ego)	—	—	—	0.112	0.026	0.000	0.021	0.026	0.436	0.089	0.034	0.009
Gender: Female (Ego)	—	—	—	0.171	0.053	0.001	0.014	0.054	0.790	0.067	0.058	0.252
Ethnicity: Miskito (Ego)	—	—	—	0.113	0.112	0.310	0.200	0.112	0.074	0.119	0.121	0.323
Melanin Index (Ego)	—	—	—	−0.080	0.029	0.006	−0.070	0.029	0.014	−0.049	0.030	0.102
Body Mass Index (Ego)	—	—	—	0.022	0.027	0.415	0.033	0.027	0.220	0.034	0.027	0.206
Age Similarity	—	—	—	0.113	0.108	0.295	0.197	0.124	0.112	0.258	0.119	0.029
Same Gender	—	—	—	0.144	0.043	0.001	0.137	0.044	0.002	0.171	0.046	0.000
Same Ethnicity	—	—	—	0.032	0.099	0.748	0.002	0.101	0.985	0.049	0.106	0.648
Melanin Index Similarity	—	—	—	−0.133	0.134	0.320	−0.058	0.153	0.702	−0.129	0.143	0.369
Body Mass Index Similarity	—	—	—	−0.251	0.158	0.110	−0.405	0.177	0.022	−0.222	0.172	0.197
Out-degree Activity	—	—	—	—	—	—	—	—	—	0.004	0.003	0.232
In-degree Popularity	—	—	—	—	—	—	0.048	0.008	0.000	0.049	0.011	0.000
Out-degree Popularity	—	—	—	—	—	—	−0.071	0.009	0.000	−0.043	0.014	0.003
Transitive Triplets	—	—	—	—	—	—	0.090	0.006	0.000	0.320	0.021	0.000
Transitive Reciprocated Triplets	—	—	—	—	—	—	—	—	—	−0.338	0.049	0.000
Three Cycles	—	—	—	—	—	—	—	—	—	0.069	0.035	0.051
Dense Triads	—	—	—	—	—	—	—	—	—	0.145	0.035	0.000
Transitive Triplets Jumping HHs	—	—	—	—	—	—	—	—	—	0.105	0.035	0.003
Shared Popularity	—	—	—	—	—	—	—	—	—	−0.018	0.002	0.000

GOF Test	MHD	GOF *p*	MHD	GOF *p*	MHD	GOF *p*	MHD	GOF *p*
In-degree Distribution	520.199	0.002	138.851	0.000	54.442	0.096	51.188	0.121
Out-degree Distribution	305.098	0.000	138.663	0.000	34.255	0.577	30.438	0.761
Distribution of Geodesic Distances	532.930	0.000	270.699	0.001	9.885	0.109	4.801	0.339
Triad Census	835.023	0.000	490.542	0.000	136.071	0.000	22.604	0.108
Clique Census	178.152	0.002	85.923	0.008	7.936	0.225	3.090	0.507
Consanguineous Ties	65.633	0.000	49.506	0.003	36.794	0.032	29.683	0.090

*p* = *p*-value (two-tailed) associated with the test statistic ${t}_{{\hat{\beta }}_{k}}$ = ${\hat{\beta }}_{k}\div{e}_{{\hat{\beta }}_{k}}$. *MHD* = Joint Mahalanobis Distance. *GOF p* = Monte Carlo MHD test *p*-value (*H*_*Null*_: Observed and simulated distribution are the *same*; GOF *p* > 0.05 is desirable; one-tailed). In-degree Range = 0–39. Out-degree Range = 0–36. Geodesic Distances Range = 1–5 & Infinity. Clique Size Range = 1–8. For details on SAOM estimation/convergence settings, see *Methods*. Results rounded to the nearest thousandth for presentation.

**Table 3:** Estimated SAOM parameters $\widehat{\beta }$ when the rate parameter *λ* is fixed at 108. Estimates are log odds ratios (e.g., ${\widehat{\beta }}_{{\rm{Reciprocity}}}=1.7$ indicates that the odds of the aid relation ${x}_{ij}$ is, conditional on the other effects, larger by a factor of 5.5 (*e*^1.7^) when *x*_*ij*_ reciprocates the tie *x*_*ji*_).

SAOM Effect	Conventional Model (5)			Extended Model (6)			Networked Aid Model (Limited) (7)			Networked Aid Model (Comprehensive) (8)		
	$\widehat{{\boldsymbol{\beta }}}$	${{\boldsymbol{s}}{\boldsymbol{e}}}_{\widehat{{\boldsymbol{\beta }}}}$	*p*	$\widehat{{\boldsymbol{\beta }}}$	${{\boldsymbol{s}}{\boldsymbol{e}}}_{\widehat{{\boldsymbol{\beta }}}}$	*p*	$\widehat{{\boldsymbol{\beta }}}$	${{\boldsymbol{s}}{\boldsymbol{e}}}_{\widehat{{\boldsymbol{\beta }}}}$	*p*	$\widehat{{\boldsymbol{\beta }}}$	${{\boldsymbol{s}}{\boldsymbol{e}}}_{\widehat{{\boldsymbol{\beta }}}}$	*p*
Rate Param. (Avg. Tie Changes)	108	Fixed	—	108	Fixed	—	108	Fixed	—	108	Fixed	—
Out-degree (Aid Arbitrary People)	−1.540	0.065	0.000	−1.660	0.124	0.000	−1.681	0.148	0.000	−2.407	0.158	0.000
Reciprocity	1.661	0.060	0.000	1.731	0.076	0.000	1.849	0.102	0.000	2.167	0.115	0.000
Relative Wealth Rank	—	—	—	0.010	0.008	0.176	0.011	0.008	0.146	0.006	0.006	0.338
Geographic Distance	−0.063	0.010	0.000	−0.114	0.015	0.000	−0.083	0.016	0.000	−0.066	0.015	0.000
Consanguineal Relatedness	2.831	0.123	0.000	3.294	0.338	0.000	2.579	0.347	0.000	1.655	0.337	0.000
Cons. Rel. × Reciprocity	—	—	—	−1.300	0.380	0.001	−1.409	0.411	0.001	−0.510	0.404	0.208
Cons. Rel. × Relative Wealth Rank	—	—	—	−0.017	0.016	0.288	−0.021	0.016	0.191	−0.012	0.016	0.426
Cons. Rel. × Geographic Distance	—	—	—	0.047	0.048	0.325	0.004	0.048	0.926	0.005	0.052	0.921
Affinal Relatedness	2.064	0.103	0.000	2.014	0.113	0.000	1.352	0.129	0.000	0.947	0.121	0.000
Godparental Relation	—	—	—	0.399	0.061	0.000	0.373	0.069	0.000	0.333	0.069	0.000
Infidelity Relation	—	—	—	−0.487	0.182	0.008	−0.388	0.188	0.039	−0.172	0.178	0.335
Household Wealth (Alter)	—	—	—	0.042	0.064	0.516	0.085	0.066	0.196	0.016	0.060	0.788
Household Size (Alter)	—	—	—	−0.004	0.022	0.858	−0.045	0.020	0.023	−0.025	0.021	0.247
Age (Alter)	—	—	—	0.072	0.021	0.001	0.115	0.029	0.000	0.045	0.032	0.163
Gender: Female (Alter)	—	—	—	0.124	0.049	0.011	0.123	0.047	0.009	0.045	0.051	0.379
Ethnicity: Miskito (Alter)	—	—	—	0.044	0.097	0.650	0.041	0.086	0.636	0.074	0.098	0.447
Melanin Index (Alter)	—	—	—	−0.061	0.025	0.015	−0.064	0.021	0.002	−0.032	0.025	0.206
Body Mass Index (Alter)	—	—	—	−0.085	0.023	0.000	−0.033	0.021	0.118	−0.053	0.023	0.021
Household Wealth (Ego)	—	—	—	0.013	0.064	0.843	−0.033	0.063	0.598	−0.011	0.054	0.844
Household Size (Ego)	—	—	—	−0.127	0.025	0.000	−0.105	0.027	0.000	−0.109	0.026	0.000
Age (Ego)	—	—	—	0.098	0.021	0.000	−0.001	0.026	0.957	0.097	0.028	0.000
Gender: Female (Ego)	—	—	—	0.170	0.048	0.000	0.037	0.052	0.479	0.095	0.049	0.052
Ethnicity: Miskito (Ego)	—	—	—	0.110	0.100	0.274	0.154	0.101	0.128	0.088	0.100	0.376
Melanin Index (Ego)	—	—	—	−0.077	0.026	0.003	−0.062	0.028	0.026	−0.040	0.024	0.099
Body Mass Index (Ego)	—	—	—	0.027	0.024	0.261	0.025	0.026	0.324	0.024	0.023	0.295
Age Similarity	—	—	—	0.054	0.079	0.496	0.048	0.108	0.657	0.190	0.088	0.030
Same Gender	—	—	—	0.131	0.032	0.000	0.131	0.035	0.000	0.162	0.036	0.000
Same Ethnicity	—	—	—	0.042	0.081	0.605	0.023	0.081	0.775	0.065	0.084	0.437
Melanin Index Similarity	—	—	—	−0.110	0.100	0.269	−0.073	0.126	0.562	−0.090	0.109	0.411
Body Mass Index Similarity	—	—	—	−0.194	0.117	0.098	−0.249	0.172	0.146	−0.192	0.124	0.123
Out-degree Activity	—	—	—	—	—	—	—	—	—	0.002	0.003	0.483
In-degree Popularity	—	—	—	—	—	—	0.047	0.009	0.000	0.045	0.009	0.000
Out-degree Popularity	—	—	—	—	—	—	−0.079	0.011	0.000	−0.041	0.013	0.002
Transitive Triplets	—	—	—	—	—	—	0.074	0.006	0.000	0.314	0.019	0.000
Transitive Reciprocated Triplets	—	—	—	—	—	—	—	—	—	−0.318	0.047	0.000
Three Cycles	—	—	—	—	—	—	—	—	—	0.058	0.034	0.088
Dense Triads	—	—	—	—	—	—	—	—	—	0.135	0.035	0.000
Transitive Triplets Jumping HHs	—	—	—	—	—	—	—	—	—	0.104	0.031	0.001
Shared Popularity	—	—	—	—	—	—	—	—	—	−0.019	0.002	0.000

GOF Test	MHD	GOF *p*	MHD	GOF *p*	MHD	GOF *p*	MHD	GOF *p*
In-degree Distribution	535.293	0.000	89.468	0.002	66.021	0.044	58.324	0.045
Out-degree Distribution	277.805	0.000	127.344	0.000	53.057	0.072	33.231	0.633
Distribution of Geodesic Distances	475.663	0.001	210.939	0.001	27.077	0.023	3.742	0.445
Triad Census	850.655	0.000	528.406	0.000	142.173	0.001	14.417	0.397
Clique Census	186.293	0.001	76.362	0.011	16.371	0.097	2.850	0.579
Consanguineous Ties	102.266	0.000	71.481	0.000	56.559	0.007	43.878	0.013

BOLD estimates differ in sign when *λ* is fixed at 36 (see Table 2). *p* = *p*-value (two-tailed) associated with the test statistic ${t}_{{\hat{\beta }}_{k}}$ = ${\hat{\beta }}_{k}\div{e}_{{\hat{\beta }}_{k}}$. *MHD* = Joint Mahalanobis Distance. *GOF p* = Monte Carlo MHD test *p*-value (*H*_*Null*_: Observed and simulated distribution are the *same*; GOF *p* > 0.05 is desirable; one-tailed). In-degree Range = 0–39. Out-degree Range = 0–36. Geodesic Distances Range = 1–5 & Infinity. Clique Size Range = 1–8. For details on SAOM estimation/convergence settings, see *Methods*. Results rounded to the nearest thousandth for presentation.

For brevity, I only discuss Table 2. And to assist readers, pictograms (“→”) indicating the arrangement of aid relationships (i.e., who helps who) between individuals (e.g., *i*, *j*, *h*, etc.) in the various scenarios captured by the network-structure-related effects are used to discuss the estimates throughout the Results section. For example, a complete (i.e., a “Simmelian” or “dense”) triad is indicated by $\left[i\rightleftarrows h\rightleftarrows j\rightleftarrows i\right]$. In pictograms such as this one, all of which correspond to the formulae in Online-Only Table 1, the focal actor — i.e., the individual whose decision to aid another network member is being modelled by the SAOM (hence, “actor-oriented”) — is always labelled “*i*”.

### Robust evidence of aid in line with kinship, reciprocity, and proximity

Regardless of model, and conditional on the other effects, estimates suggest that the log odds of creating and maintain an aid relationship *x*_*ij*_ is positively associated with: (*i*) being helped (*Reciprocity*); (*ii*) living in close proximity (*Geographic Distance*); and (*iii*) being related — whether by blood (*Consanguineal Relatedness*), marriage (*Affinal Relatedness*), or choice (*Godparental Relation*). There is no compelling evidence to suggest need-based transfers (*Relative Wealth Rank*). And evidence of a negative interaction between relatedness and reciprocity in Model 2 (${\widehat{\beta }}_{{\rm{Consanguineal}}{\rm{Relatedness}}\times {\rm{Reciprocity}}}=-1.288$, *se* = 0.407, *p* = 0.002) and Model 3 (${\widehat{\beta }}_{{\rm{Consanguineal}}{\rm{Relatedness}}\times {\rm{Reciprocity}}}=-1.180$, *se* = 0.420, *p* = 0.005) disappears after the inclusion of the remaining network-structure-related effects in Model 4 (${\widehat{\beta }}_{{\rm{Consanguineal}}{\rm{Relatedness}}\times {\rm{Reciprocity}}}=-0.675$, *se* = 0.428, *p* = 0.115). Moreover, Model 2, Model 3, and Model 4 provide no compelling evidence of interactions between need and kinship (*Consanguineal Relatedness* × *Relative Wealth Rank*; i.e., kin-directed altruism) or distance and kinship (*Consanguineal Relatedness* × *Geographic Distance*).

### Clear evidence of aid in line with manifold supra-dyadic dynamics

There is strong observational evidence of simple transitivity in Model 3 (${\widehat{\beta }}_{{\rm{Transitive}}{\rm{Triplets}}}=0.090$, $se=0.006$, $p < 0.001$) and Model 4 (${\widehat{\beta }}_{{\rm{Transitive}}{\rm{Triplets}}}=0.320$, $se=0.021$, $p < 0.001$). Of course, transitive triads may be a by-product of unobserved homophilous preferences and unobserved opportunities for interaction (see Rivera *et al*.^39^, p. 105–107, and Stadtfeld *et al*.^47^). Still, conditional on the five homophily effects and proximity, both Model 3 and Model 4 indicate that the log odds of creating and maintaining an aid relationship *x*_*ij*_ is positively associated with the presence of (*i*) indirect aid to *j* via third parties (i.e., a tendency for $i\to j$ in the transitive triad $\left[i\to h\to j\leftarrow i\right]$); and (*ii*) shared targets of aid (i.e., a tendency for $i\to j$ in the transitive triad $\left[i\to j\to h\leftarrow i\right]$). This is expected given published results from generative models of social support networks in subsistence populations^28–34^. However, Model 4, which features a more comprehensive set of network-structure-related effects compared to prior evolutionary work, indicates the operation of group dynamics over and above simple transitivity, net of simple reciprocity, homophily, proximity, and kinship.

Specifically, and in line with important research by Block^43^, Model 4 provides strong observational evidence of a negative interaction between transitivity and reciprocity (i.e., a tendency against $i\to j$ in the triad $\left[i\to h\to j\rightleftarrows i\right]$; ${\widehat{\beta }}_{{\rm{Transitive}}{\rm{Reciprocated}}{\rm{Triplets}}}=-0.338$, *se* = 0.049, *p* < 0.001). This suggest that weak ties with third parties may help stabilise the asymmetric provision of aid^43^. Concurrently, there is weak evidence to suggest that residents engage in simple chain-generalised exchange^19,20^ (${\widehat{\beta }}_{{\rm{Three}}{\rm{Cycles}}}=0.069$, *se* = 0.035, *p* = 0.051). That is, Model 4 indicates that the log odds of creating and maintaining an aid relationship *x*_*ij*_ is positively associated with *i*’s indirect receipt of support from *j* (i.e., a tendency for $i\to j$ in the cyclic triad $\left[i\leftarrow h\leftarrow j\leftarrow i\right]$).

Moreover, there is strong evidence to suggest helping across household boundaries (${\widehat{\beta }}_{{\rm{Transitive}}{\rm{Triplets}}{\rm{Jumping}}{\rm{HHs}}}=$ $0.105$, *se* = 0.035, *p* = 0.003). That is, conditional on the other effects, mixed-household two-paths $[{i}_{{\rm{HH}}-1}\to$ ${h}_{{\rm{HH}}-1}\to {j}_{{\rm{HH}}-2}]$ are positively associated with the log odds of creating and maintaining the inter-household tie ${i}_{{\rm{HH}}-1}\to {j}_{{\rm{HH}}-2}$. Thus, coresidents who help one another appear to converge on aid targets outside of their home (i.e., $\left[{i}_{{\rm{HH}}-1}\to {h}_{{\rm{HH}}-1}\to {j}_{{\rm{HH}}-2}\leftarrow {i}_{H{\rm{H-}}1}\right]$). This dynamic has been linked to the maintenance of intra-household harmony in prior evolutionary work^27^.

Further still, Model 4 points to the existence of cohesive support groups (${\widehat{\beta }}_{{\rm{Dense}}{\rm{Triads}}}=0.145$, *se* = 0.035, *p* < 0.001). More precisely, Model 4 provides strong observational evidence to suggest that the log odds of creating and maintaining an aid relationship *x*_*ij*_ is positively associated with the scenario wherein a focal resident *i* and an alter *j* from whom *i* receives aid both exchange resources with a third villager *h* (i.e., a tendency for $i\to j$ in the 3-clique $\left[i\rightleftarrows h\rightleftarrows j\rightleftarrows i\right]$). That said, Simmelian tie theory in its classic form does not posit that individuals actively create complete triads^43,48^, only that ties in complete triads are resistant to dissolution once formed. Thus, without longitudinal data to clarify, it is unclear to what extent the positive *Dense Triads* effect reflects the maintenance of old aid ties versus an impetus to begin helping others in line with the third-party integrative pressure intrinsic to Simmelian groups^48,63^.

Crucially, a proper interpretation of results from generative network models considers structural effects jointly. This is because they relate to the emergence of sub-graphs that are generally nested like matryoshki (i.e., “Russian Dolls”). For example, a single complete triad $\left[i\rightleftarrows h\rightleftarrows j\rightleftarrows i\right]$ is comprised of three reciprocated dyads, six transitive triplets, six transitive reciprocated triplets, two three cycles, and two possible inter-household transitive triplets. Thus, the signs of the “lower-order” effects in Model 4 concern the establishment of sub-graphs over and above those nested within complete triads. Accordingly, the estimates — i.e., positive *Dense Triads* alongside positive *Transitive Triplets*, negative *Transitive Reciprocated Triplets*, and positive *Three Cycles* — collectively suggest a preponderance of weakly-connected groups outside of maximally-connected regions of the tangible aid network which, in the present case, appears to help facilitate macro-level system integration (see Fig. 1).

As for degree-based dynamics, there is strong evidence to suggest that, conditional on the other effects, the log odds of creating and maintaining an aid relationship *x*_*ij*_ is negatively associated with the out-degree (i.e., activity) of *j* in Model 3 (${\widehat{\beta }}_{{\rm{Out-degree}}{\rm{Popularity}}}=-0.071$, *se* = 0.009, *p* < 0.001) and Model 4 (${\widehat{\beta }}_{{\rm{Out-degree}}{\rm{Popularity}}}=-0.043$, *se* = 0.014, *p* = 0.003). Put alternatively, there is a tendency against indirect reciprocity (i.e., $i\to j$ in the two-path $\left[i\to j\to h\right]$) outside of transitive, cyclic, and dense triads. Furthermore, there is strong evidence to suggest that the log odds of creating and maintaining *x*_*ij*_ is positively associated with the in-degree (i.e., popularity) of *j* (i.e., a tendency for $i\to j$ in the in-two-star $\left[i\to j\leftarrow h\right]$) in Model 3 (${\widehat{\beta }}_{{\rm{In-degree}}{\rm{Popularity}}}=0.048$, $se=0.008$, $p < 0.001$) and Model 4 (${\widehat{\beta }}_{{\rm{In-degree}}{\rm{Popularity}}}=0.049$, *se* = 0.011, *p* < 0.001). However, like above, and in both Model 3 and Model 4, the positive *In-degree Popularity* effect indicates a preponderance of in-stars over and above those nested within transitive and dense triads and thus more-global returns on popularity (see Fig. 1).

That said, help in Arang Dak appears to be structured in a fashion whereby aid provision is not redundant across those in need. Specifically, the positive *In-degree Popularity* effect indicates a tendency for residents to receive aid by virtue of having many sources of support. However, the negative *Shared Popularity* effect strongly evidenced by Model 4 ($\widehat{\beta }=-0.018$, *se* = 0.002, *p* < 0.001) indicates that, conditional on the other effects, the log odds of creating and maintaining an aid relationship *x*_*ij*_ is negatively associated with the scenario wherein *i* helps the same network members as one of *j*’s patrons *h*. Put alternatively, popular recipients of aid — who themselves may not help many others given the negative *Out-degree Popularity* effect — tend to have divergent donor pools (i.e., a tendency against $i\to j$ in the tetrad $\left[i\to j\leftarrow h\to k\leftarrow i\right]$; n.b., the nested in-two-stars centred on *j* and *k*).

Given new experimental findings concerning the Agta hunter-gatherers of the Philippines^8^, the signs of the three popularity effects perhaps jointly point to a kind of need-based transfer in a village characterised by wealth inequality^27^. That is, these results perhaps suggest that residents help those who, based on the concentrated altruism of others, are understood to lack sufficient means (Positive *In-Degree Popularity*; Negative *Shared-Popularity*) as opposed to helping those who are understood to be in a position to give based on their level of patronage (Negative *Out-degree Popularity*; see also Smith *et al*.^8^ on alter cooperativeness and Macfarlan, Quinlan, and Remiker^66^ on levels of patronage and prosocial reputations).

### Supra-dyadic dynamics prominently govern expected aid provision

The prior section concerns evidence for network-structure-related constraints on aid provision. This evidence is implicitly assumed to exist by my research question and was thus in need of confirmation. I now turn to values of *I*_*k*_(*x*) — i.e., the relative importance of each effect *k* under each of the four models — which appear in Table 4. Recalling the multinomial logit underlying each SAOM, *I*_*k*_(*x, i*) is the proportional contribution of the *k*th effect to the distribution of the probabilities of each possible change to one’s portfolio of outgoing ties that an actor *i* could make in departure from the *observed state* of the analysed network *x* (i.e., *x*(*t*_2013_)) given the SAOM-specific parameter estimates $\widehat{\beta }$ corresponding to the set of researcher-chosen effects *k*^41^. And *I*_*k*_(*x*) is simply the average of the actor-specific proportional contributions *I*_*k*_(*x,i*) across all *N* members of network *x*. Put alternatively, *I*_*k*_(*x*) is the global expected influence of the *k*th effect over the relational decisions of residents of Arang Dak in the hypothetical scenario wherein they are presented with the opportunity to modify the observed state of the network *x* by changing a single tie (see *Methods* for a more technical definition of *I*_*k*_(*x, i*)).

**Table 4:** Global relative importance *I*_*k*_(*x*) for estimated SAOM parameters $\widehat{\beta }$ when the rate parameter *λ* is fixed at 36 and fixed at 108.

SAOM Effect	Conventional Model		Extended Model		Networked Aid Model (Limited)		Networked Aid Model (Comprehensive)	
	*λ* = 36	*λ* = 108	*λ* = 36	*λ* = 108	*λ* = 36	*λ* = 108	*λ* = 36	*λ* = 108
	*I*_*k*_(*x*)	*I*_*k*_(*x*)	*I*_*k*_(*x*)	*I*_*k*_(*x*)	*I*_*k*_(*x*)	*I*_*k*_(*x*)	*I*_*k*_(*x*)	*I*_*k*_(*x*)
Out-degree (Aid Arbitrary People)	0.374	0.371	0.238	0.242	0.169	0.162	0.119	0.122
Reciprocity	0.289	0.291	0.186	0.190	0.131	0.139	0.108	0.110
Relative Wealth Rank	—	—	0.029	0.028	0.016	0.020	0.010	0.008
Geographic Distance	0.122	0.125	0.133	0.132	0.067	0.068	0.035	0.038
Consanguineal Relatedness	0.123	0.123	0.092	0.093	0.046	0.048	0.021	0.020
Cons. Rel. Reciprocity	—	—	0.018	0.018	0.012	0.014	0.004	0.003
Cons. Rel. Relative Wealth Rank	—	—	0.003	0.003	0.002	0.003	0.001	0.001
Cons. Rel. Geographic Distance	—	—	0.005	0.005	0.001	0.000	0.001	0.000
Affinal Relatedness	0.091	0.091	0.053	0.054	0.021	0.023	0.010	0.011
Godparental Relation	—	—	0.012	0.011	0.008	0.007	0.004	0.004
Infidelity Relation	—	—	0.002	0.003	0.001	0.001	0.000	0.000
Household Wealth (Alter)	—	—	0.012	0.009	0.008	0.012	0.004	0.002
Household Size (Alter)	—	—	0.001	0.001	0.005	0.006	0.001	0.002
Age (Alter)	—	—	0.021	0.019	0.015	0.020	0.004	0.005
Gender: Female (Alter)	—	—	0.021	0.021	0.007	0.014	0.001	0.003
Ethnicity: Miskito (Alter)	—	—	0.002	0.002	0.002	0.001	0.002	0.002
Melanin Index (Alter)	—	—	0.014	0.013	0.010	0.009	0.003	0.003
Body Mass Index (Alter)	—	—	0.019	0.018	0.005	0.005	0.006	0.005
Household Wealth (Ego)	—	—	0.003	0.002	0.005	0.004	0.004	0.001
Household Size (Ego)	—	—	0.025	0.025	0.016	0.014	0.010	0.010
Age (Ego)	—	—	0.021	0.019	0.003	0.000	0.008	0.009
Gender: Female (Ego)	—	—	0.021	0.021	0.001	0.003	0.003	0.005
Ethnicity: Miskito (Ego)	—	—	0.004	0.004	0.005	0.004	0.002	0.002
Melanin Index (Ego)	—	—	0.015	0.015	0.009	0.008	0.004	0.004
Body Mass Index (Ego)	—	—	0.004	0.005	0.004	0.003	0.003	0.002
Age Similarity	—	—	0.006	0.003	0.007	0.002	0.006	0.005
Same Gender	—	—	0.024	0.022	0.015	0.015	0.013	0.013
Same Ethnicity	—	—	0.006	0.009	0.000	0.003	0.004	0.006
Melanin Index Similarity	—	—	0.005	0.004	0.001	0.002	0.002	0.002
Body Mass Index Similarity	—	—	0.008	0.006	0.008	0.005	0.003	0.003
Out-degree Activity	—	—	—	—	—	—	0.011	0.006
In-degree Popularity	—	—	—	—	0.137	0.131	0.088	0.086
Out-degree Popularity	—	—	—	—	0.160	0.170	0.072	0.072
Transitive Triplets	—	—	—	—	0.100	0.083	0.149	0.154
Transitive Reciprocated Triplets	—	—	—	—	—	—	0.122	0.123
Three Cycles	—	—	—	—	—	—	0.025	0.022
Dense Triads	—	—	—	—	—	—	0.045	0.043
Transitive Triplets Jumping HHs	—	—	—	—	—	—	0.008	0.008
Shared Popularity	—	—	—	—	—	—	0.082	0.087

Each value *I*_*k*_(*x*) indicates the average proportional contribution of the *k*th effect (i.e., covariate) to the distribution of the probabilities of each possible change to one’s outgoing ties that some network member *i* could make in departure from the observed state of the analysed network *x* given the SAOM-specific parameter estimates $\widehat{\beta }$ in Tables 2 and 3.

Values of *I*_*k*_(*x*) for Model 1 (i.e., the Conventional Model) and Model 2 (i.e., the Extended Model) are in line with what one would expect *a priori* and based on: (*i*) previous evolutionary studies of supportive relationships in traditional human populations;^1,3,5–9,13,14,27–34^ and (*ii*) a recent meta-analysis of food sharing amongst human and non-human primates^4^. That is, under Model 1 and Model 2, *Consanguineal Relatedness* and *Reciprocity*, alongside *Geographic Distance*, appear to hold substantial sway over who residents of Arang Dak are expected to provide tangible support to. More importantly, however, is that when Model 2 is expanded to include network-structure-related constraints on alter choice in Model 3 and Model 4 (i.e., the Networked Aid Models), the relative importance of these three effects to aid provision wanes sharply. And, under Model 3 and Model 4, the effects used to capture supra-dyadic interdependence, particularly those related to popularity and non-covariate-based transitivity, are roughly as influential and, in some cases, markedly more influential than *Consanguineal Relatedness*, *Affinal Relatedness*, *Reciprocity*, and *Geographic Distance* — where interactions between *Consanguineal Relatedness* and *Reciprocity*, *Relative Wealth Rank*, and *Geographic Distance* are marginal.

Accordingly, to the extent that the signs of the network-structure-related effects in Tables 2 and 3 reflect a kind of systemic need-based aid and a willingness to establish loose-knit cooperative groups outside of one’s immediate support-clique, values of *I*_*k*_(*x*) suggest that these dynamics are the most salient to aid provision and thus the emergence of the tangible support network spanning Arang Dak. Nevertheless, recall that *I*_*k*_(*x*) is a proportion. Consequently, it is reasonable to expect to observe some shrinkage of each share of influence as the size of a SAOM specification grows. And, despite this shrinkage, *Reciprocity* remains one of the most influential effects whilst eclipsing *Consanguineal Relatedness* — a result that is in line with findings from evolutionary studies by, for example, Allen-Arave *et al*.^1^, Jaeggi and Gurven^4^, Kasper and Borgerhoff Mulder^5^, and Nolin^7^.

### Supra-dyadic dynamics are critical to system emergence

Values of *I*_*k*_(*x*) do not reveal which archetypal specification makes a “good” model of Arang Dak’s tangible aid network — i.e., a model that reproduces fundamental topological features of the observed structure^18,40,46,47,49^. As mentioned above, judging relative model quality in this manner is crucial as I wish to assess the determinants of aid in relation to the global cooperative system within which help is embedded. In this respect, Model 3 and Model 4 are not superfluous.

Specifically, consider results from tests^64^ of the joint Mahalanobis distance (MHD) between the components of distributions comprised of features of the real aid network (e.g., the number of residents with an in-degree = 0, 1, 2, etc.) and the average of these same features across 20,000 synthetic networks simulated under each model (Table 2; bottom). As Fig. 1 indicates, aid relationships in Arang Dak cohere to form very rich global structure. Thus, I gauge model quality using a diverse set of aggregated micro- and macro-level network features^40,49^. Namely, I focus on: (*i*) the distributions of the in-degrees, the out-degrees, and the geodesic distances (i.e., shortest paths); alongside (*ii*) the triad census (i.e., the frequencies of each of the 16 possible non-isomorphic sub-graphs of three villagers); and (*iii*) the clique census (i.e., the frequencies of each inclusion-maximal, completely-connected sub-graph of size *w* appearing in the observed aid network). Furthermore, for a topic-specific test, I consider: (*iv*) the distribution of the number of aid relationships amongst each observed type of consanguineal relative (i.e., coefficient of genetic relatedness = 0.5 (parent/child; full siblings), 0.25 (grandparent/grandchild), 0.125 (first cousins), …, 0.00390625 (third cousins)).

Of course, one can expect models that ignore self-organisation to perform poorly vis-à-vis the reproduction of global network structure. Accordingly, what is interesting here is precisely *how poorly* do structure-agnostic models perform relative to a model that accounts for complex interdependences. Keeping this in mind and comparing joint Mahalanobis distances *within* the set of four tests associated with each of the six distributions (Table 2; bottom), Model 3 and Model 4 are clearly superior to Model 1 and Model 2 — both of which fail to generate plausible graphs given what was observed in Arang Dak by wide margins.

Model 3, which typifies specifications used in prior evolutionary studies of social support featuring generative network models, is an improvement over Model 1 and Model 2. Still, Model 3 fails to reproduce the Triad Census. Thus, Model 3 is suspect and inferior to Model 4 despite Model 3 capturing the distribution of in-degrees, out-degrees, geodesic distances, cliques, and consanguineous ties. Note that the relatively large joint Mahalanobis distances associated with the fit of Model 1 are unsurprising as its specification is anaemic. However, the inadequacy of Model 2 is interesting given its inclusion of effects for kinship, physical proximity, trait-based activity, trait-based popularity, and homophily as these effects could, in principle, induce supra-dyadic structure alone (see Refs. ^39,47,61,62^).

SAOMs have no single figure for model selection that is equivalent to the AIC/BIC. However, one may consider the global degree of certainty *R*_*H*_ — i.e., Snijders’^44,67^ Shannon-entropy-based measure of explained variation. *R*_*H*_ is the network (i.e., global) average of the actor-specific predictability of which other network member a focal actor *i* is expected to choose as an alter in departure from the state of the observed network *x* under a fitted SAOM. *R*_*H*_ ranges from zero to one — where zero indicates complete uncertainty (i.e., a uniform probability distribution for the potential alter choices) and one indicates complete certainty (i.e., one network member has all probability mass for a given choice). However, in practice, *R*_*H*_ will be low given the high variability of chosen alters and the difficulty of predicting precisely which dyads will have ties present given the stochastic nature of the actor-oriented model^44,67^. With that in mind, *R*_*H*_ for Model 1, Model 2, Model 3, and Model 4 are, respectively, 0.154, 0.166, 0.180, and 0.214. This is further evidence that Model 4 is an improvement over Model 1, Model 2, and Model 3.

Finally, the *χ*^2^ test statistic for the multi-parameter Wald test of whether the effects added in Model 4 over those in Model 3 are all simultaneously equal to zero is 151.56 (*df* = 6; *p* < 0.005). And the *χ*^2^ test statistic for the multi-parameter Wald test of whether the effects added in Model 3 over those in Model 2 are all simultaneously equal to zero is 268.07 (*df* = 3; *p* < 0.005). This is further evidence that tangible aid in Arang Dak is associated with network-structure-related dynamics beyond simple reciprocity that operate over and above preferences for helping kin.

## Discussion

Compared to other animals, the extent to which humans cooperate, particularly with unrelated individuals, “defies biological expectation” and is “unique in its degree and its scope” (Voorhees, Read and Gabora^68^, p. 194). Consequently, clarifying who we choose to cooperate with and why — especially in naturalistic scenarios — is a major scientific task.

Explicitly adopting a sociological and sociocentric perspective, here I have tried to add to our evolutionary understanding of human cooperation by using simulations of network dynamics to analyse exceptional public data on genetic kinship and tangible aid across an entire population of adult horticulturalists. In so doing, I have provided clear, quantitative evidence around the relative importance of competing mechanisms of cooperation in a setting significant to our evolutionary past^69^. Specifically, my results indicate that relatedness and reciprocity are markedly less important to whom one helps compared to sociological dynamics intrinsic to the arrangement of the network of tangible aid itself — particularly those related to popularity-biased attachment and pure transitivity. Furthermore, my results indicate that the set of covariates typically used to explain helping behaviour vis-à-vis evolutionary theory constitute models that are incapable of generating synthetic networks with fundamental topological properties similar to those of the real tangible aid graph. Ultimately, and perhaps controversially, my findings suggest that prior evolutionary work on helping behaviour in traditional human societies may overestimate the roles of kinship and reciprocity due to overlooking several appreciable aspects of the self-organisation of social support. Thus, my findings bolster the validity of evolutionary scientists’ attempts to move beyond common, dyad-centric predictions of why humans assist each other by emphasising supra-dyadic network dynamics (e.g., see Refs. ^27–34^ and Gurven and Kraft’s response to Ready and Power^31^, p. 88–89, in addition to Hamilton *et al*.^70^). Yet my findings also suggest that these attempts, while laudable, have not gone far enough due to a narrow focus on simple transitivity.

Accordingly, evolutionary sociocentric research (cf. experiments^8,71^) intended to uncover the principal determinants of social support in traditional human societies should explicitly and comprehensively account for supra-dyadic network structure in order to accurately gauge the relative importance of kinship and reciprocity to helping behaviour. This is especially so for scholars relying on the SAOM and the ERGM as we currently lack extensive knowledge about the behaviour of estimates from these models when network-structure-related covariates are omitted and, as a result, network dependencies are ignored or incompletely represented (see especially Refs. ^40,42,43,46,47,49,61,64,65^ on the central importance of the specification of SAOMs and ERGMs).

Of course, additional studies are needed to ascertain the replicability of my findings and their generalisability to social support in other settings relevant to our evolutionary history. That said, the apportioning of salience seen here is unlikely to be universal. Indeed, in a prior study^45^ building on an ecological theory of network formation^72^, I used 162 village-specific SAOMs of friendship between rice farmers in China to show that actor-specific relative importance scores *I*_*k*_(*x,i*) can systematically vary across individuals’ physical environments — namely, *I*_*k*_(*x,i*) for the effects *Reciprocity*, *Transitive Triplets*, and *Three Cycles*. Additionally, the results I have presented here may be overdetermined by socioecological factors unique to Arang Dak.

Specifically, Amazonian horticulturalists have been found to have a higher group relatedness compared to, for example, hunter-gatherers^69,73^. Consequently, the general strength of genetic ties in communities like Arang Dak (avg. relatedness = 0.052) may itself fuel in-group cooperation^73^ and thus serve as a potent backdrop for the very rich global structure seen in Fig. 1 and, by extension, the network-structure-related dynamics evidenced by Tables 2 and 3 (see also Table 1 of Walker^73^, p. 385, and Table 1 of Koster *et al*.^74^, p. 3, to compare relatedness amongst the Mayangna and Miskito to groups in different societies). Along this line, and speculatively speaking, the higher group relatedness in Arang Dak may then depress the relative importance of consanguinity as the distinction between close and distant/non-kin becomes less relevant to any one decision about whom to help, possibly explaining the very low values of *I*_*k*_(*x*) for *Consanguineal Relatedness* and *Affinal Relatedness* in Model 3 and Model 4 compared to the values of *I*_*k*_(*x*) for network-structure-related effects such as *In-Degree Popularity*, *Transitive Triplets*, and *Shared Popularity*. Furthermore, at the time of data collection (i.e., 2013), all residents of Arang Dak were Catholic^27^. And, as Power^29^ demonstrates, religious identity can also fuel a preponderance of cooperative relationships within groups of individuals who might not otherwise be connected. Therefore, the richness of the tangible aid network and the heightened salience of the network-structure-related effects may in part stem from Arang Dak’s religious homogeneity and, as 85% of adult residents were Mayangna at the time of data collection, its ethnic homogeneity (see also Voorhees *et al*.^68^ on shared identity and the evolution of cooperation).

These characteristics of Arang Dak present an important scope condition with respect to the replicability and generalisability of my results — i.e., societies with lower group relatedness, more religious heterogeneity, and/or more ethnic heterogeneity may see network dynamics play a less prominent role in directing help. Such a possibility underscores the need for scrutiny of my observational findings through systematic comparative analyses of network formation from a socioecological perspective. And future evolutionary sociocentric research should explore fluctuation in the relative importance of kinship and reciprocity vis-à-vis other features of human communities that stand to shape cooperation such as size^45^, wealth inequality and market integration^5,31,75–77^, predominant subsistence style^45,78^, dispersal norm^28,71^, and descent rule^6^.

As for the weaknesses of my study, limitations include an inability to adjust SAOMs for: (*i*) intra-village friendship (i.e., a major conduit of social support^3,79–81^); and (*ii*) constraints on access to potential patrons beyond geographic proximity^82^. Furthermore, my focus on just one type of social support in the form of tangible aid (c.f., provision of practical knowledge^15,34,78^) is not ideal. Yet, the most severe shortcoming of my study rests with its cross-sectional design. This is because social relationships, and thus the relative importance of network dynamics, are unlikely to be static^41,51,60^ — especially in subsistence populations where resource access can be highly stochastic (see Kaplan *et al*.^83^). Accordingly, my results — which, recall, reflect the assumption that the analysed network is in short-term dynamic equilibrium — may not hold over long timescales. Given this uncertainty around temporality, future evolutionary sociocentric research should make a special effort to emulate Redhead and von Rueden^32,33^ who fit SAOMs to data on tangible aid relationships amongst Amazonian forager-horticulturalists over an eight-year period.

Finally, I conclude with two major points. First, I stress that the network-structure-related dynamics evidenced by Tables 2 and 3 are not reducible to simple reciprocity. Nor are they precluded from predominantly, or even exclusively, unfolding within sub-regions of a social support network composed entirely of close kin^1,7,14,16,28,31^ — i.e., those individuals for whom the indirect fitness benefits of cooperation will be highest and direct fitness benefits easier to realise to the extent that family members are predisposed to “trust one another, interact frequently, and rarely defect on their repayment” (Allen-Arave *et al*.^1^, p. 314–315). For example, although I found no evidence of kin-favoured reciprocity, a qualitative reading of Fig. 1 plainly indicates that cliquish sub-graphs of varying sizes saturated with close kin (i.e., consanguineal/affinal relatedness ≥ 0.125) are likely to underlie the positive and somewhat influential *Dense Triads* effect — itself a type of structurally-embedded reciprocation^48,63^. Similarly, Fig. 1 suggests that the positive and powerful *In-degree Popularity* and *Transitive Triplets* effects are at least partially underpinned at the more-local level by the 1,111 aid relationships emanating from close kin. In contrast, the other 374 aid relationships emanating from distant-kin and non-kin likely drive the more-global returns on popularity and the less-local clustering responsible for the macro-level system integration mentioned above (see also Migliano *et al*.^84^ on non-kin dyads and the integration of Agta hunter-gatherer communities). Put formally, and using the traditional three-digit M-A-N (Mutual-Asymmetric-Null) Triad classification scheme, 81% (664/820) of the observed complete triads (i.e., $\left[i\rightleftarrows k\rightleftarrows j\rightleftarrows i\right]$; M-A-N Code “300”) and 62% (623/999) of the semi-complete triads (i.e., $\left[i\rightleftarrows k\rightleftarrows j\to i\right]$; M-A-N Code “210”) are composed entirely of close kin. Yet just 39% (73/186) of the observed transitive triads (i.e., $\left[i\to k\to j\leftarrow i\right]$; M-A-N Code “030-T”) and 35% (314/897) of the in-two-stars (i.e., $\left[i\to j\leftarrow k\right]$; M-A-N Code “021-UP”) are composed entirely of close kin.

Second, it is important to state plainly that this article is not meant to be antagonistic. Specifically, I do not wish to imply that the study of cooperative mechanisms of historic interest to evolutionary scientists should be abandoned in favour of an exclusive focus on supra-dyadic network dynamics. Instead, integration of sociological and evolutionary theories of sociality has far greater scientific potential (e.g., see Tokita and Tarnita^85^) — where a major strength of the formalist study of networks within the tradition of mathematical sociology^86^ is that it is highly ecumenical. That is, scholars in this area broadly take the evidence-based view that the emergence of non-romantic social relationships in human groups is multi-mechanistic and, necessarily, multi-theoretical. Practically speaking, this implies that narrow theoretical models are unlikely to explain global network structure along several key dimensions (e.g., geodesics, triadic morphology, degree connectivity, etc.) and thus alter choice in supra-dyadic context (see Refs. ^18,39,40,42,43,46–49,51,61,64,72,81,87^ for key discussions and examples of the explanatory leverage gained via a multi-mechanistic, multi-theoretical logic).

Sceptical readers closely aligned with the evolutionary sciences may retort that being ecumenical makes the sociological study of network formation “theoretically weak” — especially in light of the elegance, strength, and trans-species relevance of predictions derived from the theories of kin selection and reciprocal altruism alone. Nevertheless, being ecumenical does not equate to being non-theoretical^86,87^ and worthy of dismissal. Again, the network-structure-related dynamics discussed here are rooted in established lines of sociological research around structural balance theory, Simmelian tie theory, social exchange theory, and structural position (see Refs. ^19,20,39,43,47–49,60–63,72,81,86–88^). This research has proved influential as formalist network thinking has percolated from sociology to the social and natural sciences more broadly. And this research — especially the strand on triads and their ability to modulate conflict — undergirds nascent, exciting evolutionary theorising around the adaptive (i.e., fitness-enhancing) value of indirect connections for humans, non-human primates, and non-human animals (see Refs. ^27,89–93^ as well as Ilany *et al*.^94,95^). Accordingly, fusion of sociological and evolutionary research strikes me as the most promising means of future theorisation and empirical investigation of the dynamics of entire social support networks to the extent that the arrangement of these systems encodes, or even fosters^33^, cooperation-relevant information that individuals can perceive and act upon.

Essential to this integrative effort will be the SAOM and the ERGM — two methods uniquely poised for multi-mechanistic analyses of cooperation due to their broad accessibility, high degree of flexibility, and explicit emphasis on interdependence across multiple scales. Of course, neither model is unimpeachable. Indeed, the SAOM is limited by its stylisation of individual behaviour (see Snijders^55^ on myopia) and the ERGM by challenges around difficult data-model combinations (see Schweinberger *et al*.^65^ on degeneracy) — where both models are computationally expensive and still under development with respect to statistical theory^42,52,54,65^. Furthermore, the intensive nature of ethnographic fieldwork — particularly for the purposes of carrying out sociometric censuses of entire subsistence populations^96,97^ — may make amassing the amount of data needed to achieve good statistical power with these models difficult^98^. And, depending on the richness of the available network data (e.g., network size, number of ties), field researchers may need to pay extra attention to model specification. Thus, the full version of the “Network Aid” model could be inappropriate, contingent on one’s inability to achieve model convergence with such a complex specification relative to one’s ability to achieve excellent convergence and good model fit with a more parsimonious set of effects.

Still, SAOMs and ERGMs are promising tools for carrying out unified relational analyses wherein researchers simultaneously test sociological and evolutionary predictions of cooperation with varying degrees of overlap^40,42,47,49,65^ — an analytic scenario that more-traditional, non-network methods may struggle with (e.g., see Getty’s response to Gurven^99^, p. 563–564). Here, I have focused narrowly on predictions derived from the theories of kin selection, reciprocal altruism, and tolerated scrounging following pioneering human evolutionary research on social support^1–10,13,14,16,27–33^. However, there are other evolutionary theories of cooperation that are relevant to explaining helping behaviour. And avenues for further exploration via sociocentric analysis and with the aid of generative network models and longitudinal data include long-term reciprocity^4,8,14,19,20^, by-product mutualism^99^, and partner choice^7,8,31,75,100^.

## Methods

### Data summary

Data from Nicaragua were collected in 2013 by Koster^27^ who released them to the public in 2018. Koster^27^ provides substantial supplementary ethnographic information (e.g., on wealth as a proxy for need). Here it suffices to say that Koster’s^27^ data on social support are especially apt for answering my research question despite being cross-sectional and concerning just one category of aid in a single village. This is due to their exceptional detail and top-quality measurement relative to data used in prior published research on social support in traditional human societies. Koster’s^27^ data are also remarkable when contrasted with the data used in research on face-to-face human social networks in advanced economies (e.g., adolescent friendship networks in school classrooms).

Specifically, the sociometric data are comprehensively annotated with information on villagers’: (*i*) attributes (i.e., age, gender, ethnicity, skin tone, BMI, and household wealth); (*ii*) familial ties (i.e., consanguineal, affinal, and social kinship); and (*iii*) spatial location (i.e., household membership and inter-household distance). Furthermore, several aspects of the reports on aid provision themselves make for unusually comprehensive relational data.

First, aid reports cover an entire adult population — not just household heads, “household representatives”, spouses, or men. Second, aid reports are not artificially censored at common cut-offs (e.g., up to 5–10 nominations). Third, aid reports are from a roster-based recognition task — not free recall — where the names on the roster were randomly presented during the sociometric interviews. Fourth, aid reports are double-sampled — i.e., the villagers reported who they habitually received tangible aid of some form from and who they habitually provided tangible aid of some form to. Fifth, aid reports were collected at the individual level — not the household level — and thus they avoid the problematic premise that households are homogenous entities that establish social ties in a unitary fashion^96^. These data-collection decisions are significant as they help mitigate measurement error which can seriously undermine network-based research^101^.

Based on my recent review of social and biomedical scientists’ measurement of face-to-face networks spanning villages in low- and middle-income countries^96^, and to the best of my knowledge of data releases in the intervening period, no other publicly available, annotated sociometric data on tangible aid of such a high quality exists. Accordingly, Koster’s^27^ data from Arang Dak are more than apt for my analysis. Indeed, they are appropriate for wide reuse across the scientific community à la Wayne W. Zachary’s^102^ classic “Karate Club” network data. This is especially so given the great difficulty of collecting face-to-face sociometric data^97^ (cf. Twitter data) concerning adults (cf. children in schools) and the relative rarity of public data on offline social networks in developing nations^103^ compared to, for example, western, educated, industrialised, rich, and democratic (WEIRD^104^) societies (e.g., the United States, the Netherlands).

For instance, Koster’s^27^ data are extremely relevant to: (*i*) sociologists and psychologists interested in modelling voluntary relationships^26,79,80^; (*ii*) ecologists and zoologists interested in contrasting social interaction across species^4,105^; (*iii*) physicists and computer scientists interested in identifying universal principles by comparing social networks to technological and biological networks^106^; and (*iv*) economists interested in the networks of the rural poor^107^. That said, those interested in re-using Koster’s^27^ dataset should note that it lacks key pieces of information in the form of variables for social closeness and friendship^3,79–81^. And note once again that Koster’s^27^ dataset is cross-sectional. Nevertheless, these two shortcomings are strongly counterbalanced by the dataset’s richness.

To learn more about life in Arang Dak and the Miskito and Mayangna, see Koster and colleagues^27,69,73,74,108–111^.

### Network measurement

To measure social support, permutations of a single question focused on tangible aid were used. Specifically, Koster^27^ (p. 6) asked: “Who provides tangible support to you at least once per month?” — where respondents were prompted with relevant examples of aid received such as firewood, food, valuable items (e.g., canoes), and help with physical tasks. Respondents were also asked the inverse. That is, villagers were invited to nominate those to whom they gave tangible support at least once per month.

In total, villagers reported 2,595 “support receiving” ties and 2,958 “support giving” ties. However, the binary, square sociomatrix *x*(*t*_2013_) representing the tangible aid network that I use to fit my SAOMs was constructed to reflect habitual resource transfer based on residents’ *mutual assent* (i.e., double confirmation). That is, *x*_*ij*_(*t*_2013_) = 1 if villager *i* reported giving tangible aid to villager *j* at least once per month **and** villager *j* reported receiving tangible aid from villager *i* at least once per month. *x*(*t*_2013_) was constructed in this manner in order to mitigate measurement error in line with authoritative sociological research on respondent accuracy^101^ (see also Ready and Power^112^ for an alternative perspective). In total, mutual assent yields 1,422 source-recipient-verified aid relationships (n.b., the product-moment graph correlation between the matrices constructed using either the reports of the receiver or the giver of aid is 0.360).

Two villagers were away during data collection. They did not provide aid reports but could be nominated by their fellow villagers. To avoid missingness, I coded ties to/from these two villagers as present/absent in line with the unilateral reports of the other 106 residents (see also Lyle and Smith^12^ for a similar approach), where the two possible ties between the two absent villagers were coded as absent. Accordingly, in total, *x*(*t*_2013_) is comprised of 1,485 asymmetric tangible aid relationships (graph density = 0.129; graph reciprocity [i.e., the proportion of arcs that are reciprocated] = 0.712; graph transitivity [weak rule] = 0.381; mean out-/in-degree = 13.75; standard deviation of out-degrees = 7.688; standard deviation of in-degrees = 7.782).

### Kinship measurement

Genealogical interviews were used to derive Wright’s coefficient of genetic relatedness (i.e., consanguineal relatedness) for each pair of residents *i* and *j* as well as the binary dyadic indicator for infidelity relations between households, the latter of which reflects information on illegitimate children. Koster^27^ (p. 10) emphasises that the use of genealogies is an appropriate proxy for genetic similarity in Arang Dak as individuals frequently disperse during early adulthood such that the population is well-mixed. Note that two residents *i* and *j* may have both a consanguineal and an affinal relation (e.g., a mother’s blood tie to her child and her affinal tie to her child via her husband). To account for this, Koster^27^ coded affinal relatedness (i.e., Wright’s coefficient of relatedness between resident *i*’s *spouse s* and resident *j*) as zero unless affinal relatedness is greater than or equal to twice the value of consanguineal relatedness for the dyad of interest. As Koster^27^ recounts, this coding decision is ethnographically valid as it reflects perceptions of kinship in Arang Dak which give primacy to marriage-based ties in the absence of stronger blood relations. In the case of multiple third-party affinal ties between *i* and *j*, affinal relatedness reflects the strongest.

### Stochastic Actor-Oriented Models versus Exponential Random Graph Models

Although it has only recently been detailed^40,42^, the cross-sectional SAOM is used here instead of the more-widely used Exponential Random Graph Model (ERGM)^42,46,49^ for two reasons. First, the SAOM has a direct measure of relative importance^41^ (discussed below). And second, the SAOM explicitly links choice between competing relationships with different rewards and costs^42^ to a scenario wherein network members’ selection of alters comprises an ever-changing, multilevel relational context for decision making^18–22^.

Like ERGMs, cross-sectional SAOMs provide insight into the self-organisation that could have given rise to the observed network despite the absence of longitudinal data — where the cross-sectionally observed network is assumed to possess traces of generative mechanisms unfolding through time (see Lusher *et al*.^49^, p.17–19). Along this line, the assumption of short-term dynamic equilibrium one must make to fit a cross-sectional SAOM is *not* radically different from that which is commonly made when using the standard (i.e., cross-sectional) ERGM. Specifically, should one use parameter estimates from an ERGM to draw micro-level conclusions about the relational behaviour of network members — e.g., interpreting the estimate for reciprocity as a bias/tendency for actors to begin/continue to respond in kind — one is forced to assume that the analysed network is in an equilibrium state (see Block *et al*.^53^, p. 183–182, for a discussion). Nevertheless, I used SAOMs due to the two reasons above.

Note that unlike the ERGM, degeneracy is generally understood to not be an issue with the cross-sectional SAOM as long as the rate parameter *λ* is fixed at a reasonable value (see Snijders and Steglich^40^, p. 243 and 265). Practically speaking, problems with degeneracy are expected for values of *λ* on the order of 10,000 or greater (see Snijders and Steglich^40^, p. 267).

Block *et al*.^42^ (p. 205–209) and Snijders and Steglich^40^ (p. 231–243) provide extensive formalism and extended details on the cross-sectional SAOM. Additionally, Block *et al*.^42^ compare cross-sectional SAOMs to ERGMs from first principles (see also Lusher *et al*.^49^, p. 130–140, and Schweinberger *et al*.^65^). For a comparison of the assumptions around temporality underlying SAOMs and ERGMs, see Block *et al*.^53^.

### SAOM estimation settings

Cross-sectional SAOMs were fit using the R programming language and the algorithm SIENA (i.e., Simulation Investigation for Empirical Network Analysis)^44^ by following the coding directive of Snijders and Steglich^40^ which requires one to randomly and arbitrarily alter a single tie in the analysed sociomatrix *x* that is used as the target network state during the simulation. This change is simply to allow the SIENA algorithm to execute as the RSIENA R package does not yet have a dedicated option for fitting the cross-sectional SAOM.

For all models presented in Tables 2 and 3, the absolute value of the convergence *t*-ratio for each effect is less than 0.1. Furthermore, the overall convergence ratio for each model is less than 0.15. Results in Table 2 and Table 3 are rounded to the nearest thousandth for presentation and obtained using four sub-phases in estimation Phase 2 (parameter values) and 20,000 iterations in Phase 3 (standard errors, convergence checks, and goodness-of-fit). Readers unversed in the SIENA algorithm and the SAOM framework should see Ripley *et al*.^44^ for a detailed discussion of these convergence diagnostics and estimation settings which were chosen to be cautious.

### The Indlekofer-Brandes measure of relative importance for SAOM effects

Formally, and as summarised in my prior work (see Simpson^45^, p. 113), the measure of Indlekofer and Brandes^41^ may be outlined as follows. For some villager *i* that has the opportunity to amend her portfolio of outgoing relations by changing a single tie variable and all other villagers *h*′ in the network *x*, let $\widehat{\beta }$ represent a set of SAOM parameter estimates that correspond to *β* — i.e., the set of effects *k* of length *L* used to specify the SAOM’s aforementioned utility function. Furthermore, and recalling the multinomial logit at the heart of the SAOM, let *π*_*i*_ represent the probability distribution implied by $\widehat{\beta }$ that assigns to each of *i*’s potential alters *j* ∈ *h*′ a value *π*_*i*_(*j*) — i.e., the probability that *i* will change the value of her tie variable with *j* by creating a tie if it does not exist or dropping a tie if it exists; all depending on the state of the network *x* — such that ${\sum }_{j=1}^{h{\prime} }{\pi }_{i}\left(j\right)=1$. For some effect *k*∈*β*, its importance is defined to be the sum of the absolute values of the pointwise differences between *π*_*i*_ and ${\pi }_{i}^{(-k)}$, the latter of which is implied by $\widehat{\beta }$ when only *s*_*k,i*_(*x*) — i.e., the actor-specific network statistic *s*_*i*_ for some effect *k* (i.e., the local-network features; e.g., see Online-Only Table 1) — is set to zero. As such, *k*’s importance is its direct contribution to *π*_*i*_ according to $\widehat{\beta }$ given *x* and thus its expected impact on *i*’s inferred relational decision (i.e., with whom will *i* modify a tie?).

This formulation of “importance” is distinct from: (*i*) the magnitude of the estimated log odds ratio ${\widehat{\beta }}_{k}$ for an effect *k*; (*ii*) the “statistical significance” of ${\widehat{\beta }}_{k}$; and (*iii*) the unstandardised network statistic *s*_*k,i*_(*x*) associated with ${\widehat{\beta }}_{k}$. Specifically, construction of *k*’s importance is akin to assessing the amount of change in a dependent variable (here, modulation of *π*_*i*_) associated with a change in some independent variable (i.e., setting *s*_*k,i*_(*x*) equal to zero). Moreover, by setting *s*_*k,i*_(*x*) equal to zero for a given actor *i* — as opposed to excluding the parameter *β*_*k*_ from a given SAOM — the complete model specification, the magnitude of parameter estimates, and the correlations between effects (as manifest in the set of estimated parameters $\widehat{\beta }$) are all respected. This would not be the case were *β*_*k*_ to be dropped and a SAOM re-estimated (see Indlekofer and Brandes^41^, p. 300–301).

Moreover, the impact of *k* on the relational decision of villager *i* given the state of the network *x*, or *I*_*k*_(*x,i*), is relative as *k*’s importance is normalised using the sum of the expected importance of all effects in *β* in order to reflect *k*’s proportional contribution to *π*_*i*_ such that $\mathop{\sum }\limits_{k=1}^{L}{I}_{k}(x,i)=1$. Thus, *I*_*k*_(*x*,*i*), and, by extension, its global average *I*_*k*_(*x*), may be compared within and across models. *This is not equivalent to comparison of the magnitude of the log odds ratios* $\widehat{\beta }$ *which can be misleading* — something clearly demonstrated in Table 2, Table 3, and Table 4 when contrasting values of *I*_*k*_(*x*) and the magnitude of the estimates $\widehat{\beta }$ within and across the models. As network change is simulated (i.e., unobserved), *I*_*k*_(*x*,*i*) and, by extension, *I*_*k*_(*x*) are based on the observed state of the analysed network (i.e., *x*(*t*_2013_)).

Readers are directed to Indlekofer and Brandes^41^ who provide extensive logic and additional formalism for their measure. However, two points merit mention here. First, comparing values of *I*_*k*_(*x*) within and across models is valid and my usage of *I*_*k*_(*x*) for this research is in line with the original application of the measure (see Indlekofer and Brandes^41^, p. 295–297). And second, a major shortcoming of the measure of Indlekofer and Brandes^41^ is that it is not designed to account for uncertainty of the estimated parameters $\widehat{\beta }$. That is, *I*_*k*_(*x*,*i*) only reflects the parameter estimates (irrespective of their standard errors), the model specification, and the observed state of the network. These limitations are not ideal. However, no other measure for the relative importance of effects in SAOMs exists at the time of writing.

Last, values of *I*_*k*_(*x*) in Table 4 — as well as the other values reported in Table 2 and in Table 3 — are exactly reproduceable across SAOM re-estimations when: (*i*) using the model specifications in Table 2/Table 3; (*ii*) using the tangible aid network as defined above; (*iii*) carrying out estimation using a single computing core (i.e., no parallel processing); and (*iv*) using the same random seed (see Ripley *et al*.^44^, p. 62, as well as the R code for this paper on GitHub). This is because values of *I*_*k*_(*x*) are derived *after* the simulation-based estimation of a given SAOM is complete. Note that estimation using a specific random seed and a single computing core are both required to ensure that results are invariant across re-estimations of the same SAOM (see Ripley *et al*.^44^ as well as the RSIENA source code for the function sienaRI() and the function sienaAlgorithmCreate()).

### Incorporating relatedness and geographic distance into the SAOM specifications

How scholars adjust their models for consanguinity and affinity varies across the scientific literature. For example, Kasper and Borgerhoff Mulder^5^ and Nolin^7^ do not include a measure of affinity in their Conventional Models whereas Hackman *et al*.^3^ do. Furthermore, some scholars use “dummy” dyadic covariates for degrees of consanguineal relatedness and degrees of affinal relatedness separately (e.g., see Refs. ^3,8,28^) or in combination by creating a single binary dyadic covariate for “close kinship” (i.e., parent/child, siblings, spouses, parents-in-law, and siblings-in-law; e.g., see Refs. ^29–33^). Here, I strongly prefer parsimony, tractability, and maximum covariate information. Accordingly, “dummy” variables are avoided. And, as a result, consanguineal relatedness and affinal relatedness appear in their continuous form in all models, resulting in just two dyadic covariates. For other continuous implementations of relatedness, see Refs. ^1,2,6,9,10,14,15,27^. Following Preciado *et al*.^113^ and again preferring parsimony, I also included geographic distance (natural log transformation after adding a constant of one) in its continuous form in all SAOMs as opposed to using “dummy” dyadic covariates for degrees of proximity (e.g., see Hackman *et al*.^3^).

### “Networked Aid” SAOM specification vis-à-vis prior evolutionary research

Self-organisation is a basic concern for sociologists of social networks^18,40,42,46,47,49^ and the comprehensive version of the Networked Aid Model includes eight structural effects to account for the breadth of sociological research on interdependence between positive-valence (i.e., not based on disliking or aggression), asymmetric social relationships (see Refs. ^19,20,39–45,47–49,60,63,81,87,113–115^ in addition to Refs. ^46,61,72^). These eight effects also regularly feature in empirical applications of the SAOM by the model’s architects^40–44,47,52,81,113–115^. And they capture: (*i*) responding in kind (*Reciprocity*); (*ii*) self-reinforcing popularity (*In-Degree Popularity*; i.e., the Matthew Effect/Preferential Attachment); (*iii*) self-reinforcing activity (*Out-Degree Activity*; i.e., the effect of cooperativeness on itself); (*iv*) the interplay between activity and popularity (*Out-Degree Popularity*); (*v*) transitive closure (*Transitive Triplets*); (*vi*) cyclic closure (*Three Cycles*); (*vii*) the interplay between reciprocity, transitive closure, and cyclic closure (*Transitive Reciprocated Triplets*); and (*viii*) the establishment of complete triads wherein all six asymmetric relationships are present (*Dense Triads*).

The comprehensive version of the Networked Aid Model also includes a special triadic effect (*Transitive Triplets Jumping HHs*) to capture the social organisation of whole human communities in line with structural balance theory vis-à-vis household membership (see Koster^27^). And it includes a special tetradic effect (*Shared Popularity*) to account for expected deviation from structural equivalence in helping behaviour (i.e., distinction between individuals’ egocentric networks of outgoing ties) within and across households (see Simpson^96^). Note that a minimally comprehensive (cf. simply “minimal”) set of triadic effects includes *Transitive Triplets*, *Transitive Reciprocated Triplets*, *Three Cycles*, and, possibly, *Dense Triads* given sociological scholarship on social exchange theory, structural balance theory, and Simmelian tie theory; where, again, *Transitive Triplets Jumping HHs* reflects the anthropological findings of Koster^27^ vis-à-vis structural balance theory.

To the best of my knowledge, Redhead and von Rueden^32,33^ introduced SAOMs to evolutionary anthropology and human behavioural ecology with their studies on tangible support amongst the Tsimané of Bolivia. Accordingly, my analysis is only the third application of SAOMs for the purposes of testing evolutionary predictions around the formation of cooperative relationships in traditional human societies (see also Snijders *et al*.^114^ who use SAOMs to investigate social support in seven Senegalese villages with no reference to evolutionary theory). That said, Redhead and von Rueden^32,33^ only analyse aid between men. Furthermore, out of the ten network-structure-related dynamics captured in my models, Redhead and von Rueden^32,33^ only tested for five — i.e., simple reciprocity, self-reinforcing popularity, shared popularity, self-reinforcing activity, and simple transitivity using the popular geometrically-weighted edgewise shared partners effect (see Ripley *et al*.^44^, p. 44–45, 128, and 131–134, on the *gwespFF* effect versus the broader *Transitive Triplets* effect).

On the other hand, and again to the best of my knowledge of published research broadly falling under the umbrella of “evolutionary anthropology and human behavioural ecology”, Exponential Random Graph Models have been used by human evolutionary scientists at least since Nolin’s^7^ 2011 analysis of food sharing amongst the Lamaholot of Indonesia and Henrich and Broesch’s^15^ 2011 analysis of advice giving across small-scale populations in Fiji. Since the studies by Nolin^7^ and Henrich and Broesch^15^, ERGMs have featured in analyses of helping behaviour amongst the Hadza of Tanzania^2^, the Tamils of South India^28–30^, the Inuit of Canada^31^, the Tsimané^33^, and residents of small-scale populations on the Solomon Islands^34^. However, in these studies, ERGMs are only used to test for truncated degrees at zero (i.e., network isolates), self-reinforcing activity, simple reciprocity, and simple transitivity (i.e., the geometrically-weighted *edgewise* shared partners effect plus the geometrically-weighted *dyadwise* shared partners effect^29^ and, unusually, an effect for intransitivity^34^). Fisher *et al*.^37^ and Evans *et al*.^36^ review the history of SAOMs and ERGMs, respectively, across the broader behavioural ecology literature on animal social networks.

Note that there is no one-to-one correspondence between effects in SAOMs and effects in ERGMs. This is because the models use rather different dependence hierarchies — i.e., the set of assumptions around how ties feedback upon themselves. Furthermore, they reflect distinct assumptions about how social ties come into being — where ERGMs treat ties as “costless” and do not explicitly position tie formation as the result of individual decisions. That said, as implied in my comparison with SAOMs above, ERGMs are compatible with agency-based theories^42,49^.

Finally, recall that formulae for the statistics underpinning all SAOM effects used here, in addition to short descriptions to aid interpretation, appear in Online-Only Table 1. Ripley *et al*.^44^ also provide diagrams for effects that readers may find useful. Note that the interaction between *Consanguineal Relatedness* and *Reciprocity* is handled “internally” using the non-centred SAOM effect “XRecip” (Ripley *et al*.^44^, p. 140). However, the interaction between *Consanguineal Relatedness* and *Relative Wealth Rank* and the interaction between *Consanguineal Relatedness* and *Geographic Distance* are handled “externally” by multiplying the matrix for genetic relatedness with the matrix encoding the pairwise differences in wealth rank and the matrix encoding inter-household distance. These matrix products are then included in models using the non-centred SAOM effect “X” (Ripley *et al*.^44^, p. 140).

### A note on “Reciprocity”

Recall that in a SAOM the formation of relationships and, by extension, the emergence of global network structure are rooted in the decisions of individual network members (hence, “actor-oriented”). Thus, for graphs composed of asymmetric relationships, network members can only control their outgoing ties. Accordingly, the *Reciprocity* effect concerns how receiving aid impacts one’s own propensity to give back (i.e., “reciprocal exchange” à la social exchange theory^19^ or “dyadic symmetry” à la structural balance theory^48^). As von Rueden *et al*.^33^ (p. 4) highlight, this is very different from the *evolutionary strategy* of “direct reciprocity” — i.e., helping someone and judging, with some probability, that the recipient of aid will return the favour in the future (see Allen-Arave *et al*.^1^ on reciprocal altruism and “contingent reciprocity”).

In this respect, the *Reciprocity* effect in and of itself only reveals whether there tends to be a direct payoff to asymmetric aid in the form of an in-kind response by those who have been helped — where, by design, SAOMs cannot tell us if this response is the immediate result of the evolutionary strategy of reciprocity. Nevertheless, to the extent that such a strategy is employed in a given population, SAOMs can hint at its usefulness. This is also the case for the *Out-Degree Popularity* effect and the evolutionary strategy of “indirect reciprocity” — i.e., helping others to increase one’s reputation as “cooperative” in order to benefit from the greater receipt of help from others in the future (see Smith *et al*.^8^, Simpson *et al*.^20^, and Melamed *et al*.^88^).

### Network Diagram Construction

Figure 1 was created using Brandes and Wagner’s^116^ layout software *Visone*. Following Stadtfeld *et al*.^115^, the node layout was determined using Nocaj, Ortmann, and Brandes’^117^ network backbone algorithm which is designed to sparisfy graphs using a criterion based on the embeddedness of ties with the aim of increasing visual interpretability without degrading representation of meso- and macro-level network structure. The cut-off for the normalised weights used for the link sparsification behind node placement was set to 40%, where these weights are based on the embeddedness of ties in tetrads (see Nocaj *et al*.^117^ on the “quadrilateral Simmelian backbone”). The isolated node in the bottom left of the plot is for a villager who sends and receives zero ties in the source-recipient-verified tangible aid network. Note that no manual post-processing of any kind was performed when constructing the network diagram. That is, the network layout depicted in Fig. 1 was fully determined by the algorithm of Nocaj *et al*.^117^ as implemented in *Visone*^116^.

### Ethics

This paper describes the secondary analysis of public data released by Koster^27^. No primary data collection was carried out for this research. The collection of the data re-analysed here was approved by the Institutional Review Board of the University of Cincinnati^27^. Koster’s^27^ data were collected with Informed Consent^27^.

### Custom Code

Research for this paper was carried out using the open-source programming language R and widely-used R packages — all of which are detailed in the R script written for data transformation and data analysis.

## Data Availability

All data used for my analysis is available via GitHub: https://github.com/cohensimpson/smallnet_ScientificData. Permanent access to an archived version of these data is available via Zenodo^118^.

## Online-only Table

**Online-only Table 1:** Formulae for statistics *s*_*k,i*_(*x*) associated with SAOM effects.

Effect.	Verbal Description of Network Dynamic (Do villagers tend to…)	*s*_*k,i*_(*x*) = … ^§*^
Out-degree	… help arbitrary others? [*Akin to an intercept*.]	*x*_*i+*_
Reciprocity	…respond in kind when helped?	${\sum }_{j}{x}_{ij}{x}_{ji}$
Relative Wealth Rank	…help those who are comparatively less wealthy?	${\sum }_{j}{x}_{ij}\left({w}_{ij}\right)$
Geographic Distance	…help those who live further away?	
Consanguineal Relatedness	…help those who are closer genealogically?	
Affinal Relatedness	…help those who are closer genealogically through marriage?	
Godparental Relation	…help socio-religious kin?	
Infidelity Relation	…help those who they are associated with due to illegitimate children?	
Consanguineal Relatedness$\times$ Reciprocity	…respond in kind when helped by those who are closer genealogically?	${\sum }_{j}{x}_{ij}{x}_{ji}\left({w}_{ij}^{Relatedness}\right)$
Consanguineal Relatedness$\times$ Relative Wealth Rank	…help kin who are comparatively less wealthy?	${\sum }_{j}{x}_{ij}\left({w}_{ij}^{Relatedness}\times {w}_{ij}^{Relative\,Wealth\,Rank}\right)$
Consanguineal Relatedness$\times$ Geographic Distance	…help kin who live further away?	${\sum }_{j}{x}_{ij}\left({w}_{ij}^{Relatedness}\times {w}_{ij}^{Distance}\right)$
Household Wealth (Covariate-Related Activity/Ego)	…help more individuals when they have more resources?	${v}_{i}\sum _{j}{x}_{ij}$
Household Size (Covariate-Related Activity/Ego)	…help more individuals when they have more adult coresidents?	
Age (Covariate-Related Activity/Ego)	…help more individuals when they are older?	
Gender: Female (Covariate-Related Activity/Ego)	…help more individuals when they are a woman?	
Ethnicity: Miskito (Covariate-Related Activity/Ego)	…help more individuals when they are Miskito?	
Melanin Index (Covariate-Related Activity/Ego)	…help more individuals when they have a darker skin tone?	
Body Mass Index (Covariate-Related Activity/Ego)	…help more individuals when they are physically larger?	
Household Wealth (Covariate-Related Popularity/Alter)	…have more sources of aid when they have more resources?	${\sum }_{j}{x}_{ij}{v}_{j}$
Household Size (Covariate-Related Popularity/Alter)	…have more sources of aid when they have more adult coresidents?	
Age (Covariate-Related Popularity/Alter)	…have more sources of aid when they are older?	
Gender: Female (Covariate-Related Popularity/Alter)	…have more sources of aid when they are a woman?	
Ethnicity: Miskito (Covariate-Related Popularity/Alter)	…have more sources of aid when they are Miskito?	
Melanin Index (Covariate-Related Popularity/Alter)	…have more sources of aid when they have a darker skin tone?	
Body Mass Index (Covariate-Related Popularity/Alter)	…have more sources of aid when they are physically larger?	
Same Gender	…help those with the same gender?	${\sum }_{j}{x}_{ij}I\left\{{v}_{i}={v}_{j}\right\}$
Same Ethnicity	…help those in the same ethnic group?	
Age Similarity	…help those who are of a similar age?	${\sum }_{j}{x}_{ij}\left(si{m}_{ij}^{v}-{\widehat{sim}}^{v}\right)$
Melanin Index Similarity	…help those who are of a similar skin tone?	
Body Mass Index Similarity	…help those who are of a similar physical size?	
Out-degree Activity	…provide help when aiding many others?	${x}_{i+}({x}_{i+})$
In-degree Popularity	…help others with many sources of aid?	${\sum }_{j}{x}_{ij}\left({x}_{+j}\right)$
Out-degree Popularity	…help those who provide aid to many others?	${\sum }_{j}{x}_{ij}\left({x}_{j+}\right)$
Transitive Triplets	…directly help indirect recipients of aid?	${\sum }_{j,h}{x}_{ij}{x}_{ih}{x}_{hj}$
Transitive Reciprocated Triplets	…respond in kind when helped by an indirect recipient of aid?	${\sum }_{j,h}{x}_{ij}{x}_{ji}{x}_{ih}{x}_{hj}$
Three Cycles	…help those from whom they have indirectly received aid?	${\sum }_{j,h}{x}_{ij}{x}_{jh}{x}_{hi}$
Dense Triads (Complete/Simmelian Triads; 3-Cliques)	… respond in kind when helped by an exchange partner’s exchange partner?	${\sum }_{j,h}{x}_{ij}I\left\{\left({x}_{ij}+{x}_{ji}+{x}_{ih}+{x}_{hi}+{x}_{jh}+{x}_{hj}\right)=6\right\}$
Transitive Triplets Jumping Households *v*	…directly help non-coresidents helped by coresidents in receipt of aid?	${\sum }_{j\ne h}{x}_{ij}{x}_{ih}{x}_{hj}I\left\{{v}_{i}={v}_{h}\ne {v}_{j}\right\}$
Shared Popularity	…copy others by jointly supporting the same members of their community?	$\frac{1}{4}{\sum }_{j,k,h;{\rm{all}}{\rm{different}}}{x}_{ij}{x}_{hj}{x}_{ik}{x}_{hk}$

To interpret this table, note that *x*_*ij*_ = 1 indicates the presence of a tie from actor *i* to actor *j* and *x*_*ij*_ = 0 indicates the absence of a tie. *This is the same for x*_*ji*_ = 1, *x*_*ih*_ = 1, etc.

${x}_{i+}={\sum }_{i}{x}_{ij}$ = the out-degree of actor *i* and ${x}_{+i}={\sum }_{j}{x}_{ji}$ = the in-degree of actor *i*. *This is the same for x*_*j*+_ and *x*_+*j*_.

*v*_*i*_ = the value of the monadic covariate of interest for actor *i*. *This is the same for v*_*j*_.

${\widehat{sim}}^{v}$ = mean of all similarity scores for all dyads, where$si{m}_{ij}^{v}=\frac{\Delta -\left|{v}_{i}-{v}_{j}\right|}{\Delta }$ and$\Delta =ma{x}_{ij}\left|{v}_{i}-{v}_{j}\right|$ = the observed range of the covariate *v*.

The indicator function$I\left\{{v}_{i}={v}_{j}\right\}$ is equal to 1 if actor *i* and actor *j* have the same value for a categorical covariate and equal to 0 otherwise.

The indicator function$I\left\{{v}_{i}={v}_{h}\ne {v}_{j}\right\}$ is equal to 1 if actor *h* and actor *j* are *not* members of the same household and equal to 0 otherwise.

The indicator function$I\left\{\left({x}_{ij}+{x}_{ji}+{x}_{ih}+{x}_{hi}+{x}_{jh}+{x}_{hj}\right)=6\right\}$ is equal to 1 if all six ties between the actors *i*, *j* and *h* are present and equal to 0 otherwise.

*w*_*ij*_ =  the value of the dyadic covariate of interest that summarises some non-network-structure-related association between actor *i* and actor *j*.

## Code availability

All R code used for my analysis is available via GitHub: https://github.com/cohensimpson/smallnet_ScientificData. Permanent access to an archived version of this code is available via Zenodo^118^.
